# Three-dimensional numerical analysis of flow and heat transfer of bi-directional stretched nanofluid film exposed to an exponential heat generation using modified Buongiorno model

**DOI:** 10.1038/s41598-022-13351-6

**Published:** 2022-06-16

**Authors:** Wahib Owhaib, Wael Al-Kouz

**Affiliations:** Department of Mechanical and Maintenance Engineering, German Jordanian University, Amman, 11180 Jordan

**Keywords:** Engineering, Mathematics and computing

## Abstract

The heat transfer characteristics of copper/water nanofluid flow over a bi-directional stretched film are theoretically studied. The used mathematical model accounts for nanofluid effective dynamic viscosity and thermal conductivity. The model of the current study utilizes the modified Buongiorno model to scrutinize the effect of haphazard motion, nanoparticles' thermo-migration, and effective nanofluid properties. 3D flow is driven by having the nanofluid film elongation in two directions. The thermal analysis of the problem considers the nonlinear internal heat source and Newton heating conditions. In modeling the problem, the Prandtl boundary layer approximations are employed. Moreover, the nonlinear problem set of governing equations for investigating the transport of water conveying copper nanoparticles was non-dimensionalized before being treated numerically. The current parametric study investigates the impact of governing parameters on nanoparticles velocities, temperature, and concentration distributions. The presence of copper nanoparticles leads to a higher nanofluid temperature upon heating. The temperature enhances with the nanoparticles Brownian movement and thermo-migration aspects. Furthermore, involving a heat source phenomenon augments the magnitude of the heat transfer rate. Moreover, the velocity ratio factor exhibits decreasing behavior for *x-*component velocity and increasing behavior for *y-*component velocity. In conclusion, the study results proved that for larger values of *Nb* and *Nt* the temperature is higher. In addition, it is clear from the investigations that the Lewis number and Brownian motion factor decline the nanoparticle concentration field.

## Introduction

The engineered nanofluids are created by adding tiny nano-sized solid particles to traditional working fluids including water, air, oil, and ethylene glycol. Nanofluids are normally composed of metallic (Cu, Al, Au), non-metallic (Al_2_O_3_, TiO_2_, ZnO_2_), and carbon (diamond, nanotubes) oxides which have significantly improved thermal performance in various systems. Certainly, the traditional operating liquids possess a weaker thermal conductivity which can be momentously augmented by suspending nanoparticles. Because of nanofluids' remarkable thermal performance, nanofluids are useful in many industrial and technological fields comprising heating and cooling problems.

Choi and Eastman^1^ in 1995 investigated using tiny solid particles in water and established a new material that had more noteworthy thermal efficiency than pure water. Wang and Xu^2^ proved that the thermal conductivity is enhanced when adding $\mathrm{CuO}$ and ${\mathrm{Al}}_{2}{\mathrm{O}}_{3}$ nanoparticles into operating liquids including ethylene glycol, water, and engine oil. Since then, results in the literature show an improvement in heat transfer using nanoparticles. Hence, the fact of using nanofluids becomes more and more common in many industrial applications as nanofluids provide superior thermophysical properties. Furthermore, multiple physical phenomena including magnetic body forces, chemical reactions, and high-temperature behavior become more and more the feature of the emerging nanofluids applications. Thus, researchers have extensively investigated engineering and practical applications utilizing such enhanced fluids.

Al-Kouz et al.^3–7^ presented several studies on the use of nanofluids in applications related to electronic equipment cooling and heat exchangers. Al-Kouz et al.^3^ numerically investigated the flow and heat transfer characteristics of Al_2_O_3_ gaseous nanofluid at low-pressure inside a square cavity with two attached solid fins. laminar natural convection heat transfer characteristics of with entropy generation optimization studied in Al-Kouz et al.^4^. The gaseous rarefied nanofluid flow of laminar forced convection heat transfer in the entrance region of pipes examined by Al-Kouz et al.^5^. Mahanthesh et al.^6^ numerically studied Al_2_O_3_-H_2_O nanoliquid two-phase flow over a vertical flat plate under the influence of magnetic and radiation fields. Alshare et al.^7^ studied a periodically fully developed nanofluid transport through a wavy module. Rashidi et al.^8^ studied condensation characteristics of nanofluids inside smooth/rough nanochannels. Moreover, Rashidi et al.^9^ investigated the hybrid Al_2_O_3_-Cu-H_2_O nanosuspension within a lid-driven heated square chamber with a horizontal magnetic field. Mukhtar et al.^10^ provided a numerical comparison of two different nanofluids (non-Newtonian Maxwell nanofluid copper–water and molybdenum disulfide nanofluids) under unsteady magnetohydrodynamic (MHD) boundary layer flow over a porous stretching surface. Abu-Libdeh et al.^11^ numerically studied the natural convection and total entropy in a cavity under a constant magnetic field filled with Ag/MgO/H_2_O nanofluids and porous media.

In addition, nanofluids flow characteristics are of great importance when designing electromagnetic micro-pumps for the hemodialysis and lungs-on-chip devices for the pumping of the blood. Bhatti et al.^12^ examined the effect of MHD and radiation fields, and chemical reaction parameters on gyrotactic microorganisms viscous nanofluid flow in a stretched porous cylinder. Tripathi et al.^13^ conducted a thermal analysis of Cu-CuO/blood nanofluids flow in asymmetric microchannel propagating with wave velocity having the effect of microrotation effects of blood flow, thermal radiation effects, nanoparticle shape, and the effect of the electromagnetic field on the flow.

The flow of stretching nanofluid surfaces attracts researchers' attention due to the viable large number of applications as in the extrusion of plastic sheets, production of paper, condensation process of the liquid film, and glass blowing. Waqas et al.^14^ investigated Darcy–Forchheimer nanoliquid flow over the stretched surfaces on cylinder/plate considering the modified heat and mass fluxes, activation energy, and gyrotactic motile microorganism features. Zhang et al.^15^ studied the magnetic nanofluid dynamics along with a nonlinear porous stretching sheet with Arrhenius chemical kinetics and wall transpiration. They incorporated the magnetic body forces, chemical reactions, and high-temperature behavior.

There are two preferences in modeling the governing equations for the nanofluids flow problem. Hence, the heat transport of fluids conveying tiny solid nanoparticles is largely studied using one of the following two theoretical models:

1. Single-phase nanofluid model or Khanafer-Vafai-Lightstone (KVL) model^16^.
2. Two-component nanofluid model or Buongiorno nanofluid model^17^.

In the KVL model^16^, the liquid and solid phases flow with the same local velocity and they are in a thermal equilibrium state. In other words, as far as dynamics are concerned both nanoparticles and liquid particles have similar properties such as velocity, temperature, and concentration but they possess different thermo-physical properties. Thus, the nanofluid performs more like a single-phase liquid than a multi-phase solid–liquid mixture. Essentially, the main challenge in studying the nanofluid flow problem using the KVL model is incorporating the effectual properties of nanofluid. In general, nanofluid's effective properties, including the effective density, electrical conductivity, specific heats, and coefficients of thermal and solute expansions are calculated utilizing the effective average theory. However, the effective thermal conductivity and dynamic viscosity are estimated using phenomenological laws. In 1906, Einstein^17^ was the first to develop a correlation for dynamic viscosity of water conveying solid spherical nanoparticles of the volume fraction 2%. There are various models are developed to estimate the nanofluid's effective dynamic viscosity and thermal conductivity. Mishra et al.^18^ presented a review paper on theoretical models of nanofluids viscosity. They highlighted the significant effects of nanoparticles’ shape and size, temperature, volume concentration, pH, etc. Moreover, Aybar et al.^19^ revealed that nanofluids’ thermal conductivity enhancement consists of four major mechanisms: Brownian motion of the nanoparticle, nanolayer, clustering, and the nature of heat transport in the nanoparticles. They emphasize the important factors that affect the thermal conductivity modeling of nanofluids concluded in particle volume fraction, temperature, particles size, pH, and the size and property of nanolayer.

Wen and Ding^20^ experimental work proved that the use of a single-phase nanofluid model may be inadequate in cases where the friction between solid particles and liquid, thermo-migration, and haphazard movement of nanoparticles, gravity, dispersion, and sedimentation are imperative. Later, Buongiorno^21^ introduced a two-component nanofluid model covering two out of seven slip mechanisms including nanoparticles' haphazard movement and thermo-migration. By Implementing the two-component nanofluid Buongiorno model, Kuznetsov and Nield^22^ found in their study of nanofluid natural convection flow over a vertical plate that the thermal characteristics are improved by the nanoparticles' haphazard movements. Nield and Kuznetsov^23^ extended the problem of Minkowycz using Buongiorno's two-component nanofluid model and porous medium. They found that the thermo-migration of nanoparticles is positively related to the thermal boundary layer structure. The Kellerbox numeric solutions are reported by Khan and Pop^24^ for the problem of dynamics of nanofluid over an elongated plate. Makinde and Aziz^25^ extended Khan and Pop^24^ work by accounting for the Newton boundary condition. They disclosed that the strength of Newton heating has a momentous control on the thermal boundary structure. Since then, abundant research on nanofluids 2D and 3D flow utilizing the Buongiorno Model (BM) performed as follows. Gorla et al.^26^ utilized BM to study the natural convective heat transfer from a vertical stretching sheet. Khan et al.^27^ investigated 3D nanofluid flow over a bi-directional stretching sheet using BM. Gireesha et al.^28^ used BM to model Eyring-Powell fluid in two lateral directions over a convectively heated stretching sheet. Hayat et al.^29,30^ presented a numerical investigation of the 3D viscous nanofluid flow over stretchable surfaces. Mahanthesh et al.^31,32^ presented a mathematical model of nanofluid flow over an exponentially stretching sheet and non-Newtonian nanofluid flow over a stretching flat plate. Oyelakin et al.^33^ studied the 3D tangent hyperbolic nanofluid flow over a stretched sheet.

The above-mentioned studies reveal that the handling of convective heat transport characteristics using the Buongiorno model as described by the previous researchers^22–33^ has fundamentally become similar to a heat and mass transfer problem with the thermodiffusion aspect. This is insufficient as the nanoparticles are recognized to vary the thermophysical properties of nanofluids. Therefore, in the current study, it is intended to incorporate the nanofluid's effectual thermo-physical properties into Buongiorno nanofluid model. This model in the literature is known as the modified Buongiorno nanofluid model (MBM) and it was used by Yang et al.^34^, Malvandi et al.^35^, Malvandi and Ganji^36^, and many others.

The modified Buongiorno nanofluid model mathematical formulation describes the impact of volume fraction, thermophoresis, and Brownian motion on the flow characteristics.

Modeling of nanofluids using the modified Buongiorno model for many practical problems found in the literature. Puneeth et al.^37^ investigate the chemically reacting rGO-Fe_3_O4-TiO_2_-H_2_O ternary nanofluid jet flow in the presence of bio-active mixers utilizing the nanofluids modified Buongiorno model. Moreover, Malvandi et al.^35^ scrutinized the fully developed mixed convection flow of nanofluids in a vertical annular pipe utilizing the mentioned model. Furthermore, the same model is used by Khan et al.^38^ to examine the nanofluid Blasius flow with surface heat and mass fluxes. In addition to the previous applications mentioned, this model can be found in other engineering applications. For instance, Chu et al.^39^ analyzed flow due to stretching disks in presence of gyrotactic microorganisms using the model. Furthermore, the same model is applied by Alblawi et al.^40^ to solve for the flow characteristics in stretching curved surfaces. In conclusion, Owhaib et al.^41^ utilized the modified Buongiorno nanofluid model to investigate the radiation parameter influence on the 3D nanofluid rotating flow having viscous heating and prescribed heat flux. Al-Kouz & Owhaib^42^ utilized the Buongiorno nanofluid model studied non-Newtonian Casson nanofluid 3D flow with viscous heating over a linearly stretching flat surface in the rotating frame.

The current paper investigates the three-dimensional nanofluid flow and heat transfer characteristics of a bi-directional stretched nanofluid film utilizing the modified Buongiorno model. The modified Buongiorno model contains the influence of haphazard movement and thermo-migration of nanoparticles along with effective thermophysical properties. The impacts of exponential heat generation and Newton boundary conditions are examined. The modeled nonlinear partial differential boundary value problem is solved numerically, and the results are analyzed. Furthermore, A parametric study is conducted of the influence of main parameters and dimensionless numbers' on the studied system, finding presented in graphs and discussed.

## Mathematical formulation

**Figure 1:**
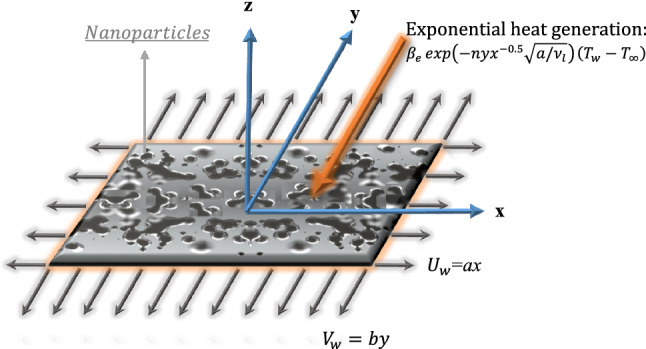
Schematic diagram of the considered problem.

A three-dimensional, steady-state boundary layer transport of water-based copper nanofluid over a bi-directional stretched surface is considered. The no-slip and Newton boundary conditions are included. The nanofluid film is stretched in two lateral directions with the velocities ${U}_{w}\left(x\right)=ax$ and ${V}_{w}(y)=by$ along $x$- and $y$- directions respectively and keeping the origin fixed. The two-component Buongiorno model is modified by including effective nanofluid properties. The nanofluid film is maintained at the concentration ${C}_{w}$ and the surface temperature ${T}_{f}$ while ${C}_{\infty }$ and ${T}_{\infty }$ are the ambient nanoparticle concentration and ambient temperature respectively. Figure 1 shows the schematic diagram of the current considered problem. The flow chart of the solution method utilized to solve the current study boundary value problem is shown in the appendix. The governing equations are as follow (Refs.^21,43^):

Conservation of mass:

1 $$\frac{\partial u}{\partial x}+\frac{\partial v}{\partial y}+\frac{\partial w}{\partial z}=0.$$

Conservation of linear momentum along *x*-direction:

2 $${\rho }_{nl}\left(u\frac{\partial u}{\partial x}+v\frac{\partial u}{\partial y}+w\frac{\partial u}{\partial z}\right)={\mu }_{nl}\frac{{\partial }^{2}u}{\partial {z}^{2}}.$$

Conservation of linear momentum along *y*-direction:

3 $${\rho }_{nl}\left(u\frac{\partial v}{\partial x}+v\frac{\partial v}{\partial y}+w\frac{\partial v}{\partial z}\right)={\mu }_{nl}\frac{{\partial }^{2}v}{\partial {z}^{2}}.$$

Conservation of energy:

4 $${\left(\rho {C}_{p}\right)}_{nl}\left(u\frac{\partial T}{\partial x}+v\frac{\partial T}{\partial y}+w\frac{\partial T}{\partial z}\right)={k}_{nl}\frac{{\partial }^{2}T}{\partial {z}^{2}}+{\left(\rho {C}_{p}\right)}_{np}\left\{{D}_{B}\frac{\partial T}{\partial z}\frac{\partial C}{\partial z}+\frac{{D}_{T}}{{T}_{\infty }} {\left(\frac{\partial T}{\partial z}\right)}^{2}\right\}+\left({T}_{f}-{T}_{\infty }\right){\beta }_{e}\mathit{exp}\left(-ny{x}^{-0.5}\sqrt{\frac{a}{{\nu }_{l}}}\right).$$

Conservation of nanoparticle concentration:

5 $$u\frac{\partial C}{\partial x}+v\frac{\partial C}{\partial y}+w\frac{\partial C}{\partial z}={D}_{B}\frac{{\partial }^{2}C}{\partial {z}^{2}}+\frac{{D}_{T}}{{T}_{\infty }}\frac{{\partial }^{2}T}{\partial {z}^{2}},$$

where $u, v$ and $w$ are the velocities components along $x, y$ and $z$*‐*directions, $\nu =\frac{\mu }{\rho }$ is the kinematic viscosity, $\mu$ is the dynamic viscosity, $\rho$ is the density, $T$ is the temperature, $C$ is the nanoparticle volume fraction, $\alpha =\frac{k}{\rho {C}_{p}}$ is the thermal diffusivity, $k$ is the thermal conductivity, ${C}_{p}$ is the specific heat, ${D}_{B}$ is the coefficient of Brownian diffusion, ${D}_{T}$ is the coefficient of thermo-migration diffusion, ${\beta }_{e}$ is coefficient of exponential space-related heat source, $a$ is a constant in the heat sourse equation, and $n>0$ is exponential index. The pertinent boundary conditions are:

6 $$\left.\begin{array}{c}v={V}_{w}(y)=by, u={U}_{w}(x)=ax, -{k}_{nl}\left(\frac{\partial T}{\partial z}\right)={h}_{f}\left({T}_{f}-{T}_{\infty }\right), C={C}_{w} at z=0, \\ \\ v=0, u=0, T={T}_{\infty }, C={C}_{\infty } as z\to \infty \end{array}\right\}$$

The effective density ${\rho }_{nl}$ and water-based *Cu* nanofluid specific heat capacity ${\left({\rho C}_{p}\right)}_{nl}$ obtained as follows:

$${\rho }_{nl}=\left(1-\phi \right){\rho }_{l}+\phi {\rho }_{np} ,$$

7 $${\left({\rho C}_{p}\right)}_{nl}=\left(1-\phi \right)(\rho {{C}_{p})}_{l}+\phi (\rho {{C}_{p})}_{np }.$$

Brinkman's dynamic viscosity model and Maxwell’s thermal conductivity model are used.

8 $$\frac{{\mu }_{nl}}{{\mu }_{l}}=\frac{1}{{\left(1-\phi \right)}^{2.5}},$$

9 $$\frac{{k}_{nl}}{{k}_{l}}=\frac{{k}_{np}+2{k}_{l}-2\phi \left({k}_{l}-{k}_{np}\right)}{{k}_{np}+2{k}_{l}+\phi \left({k}_{l}-{k}_{np}\right)}$$

the subscripts $l, nl,$ and $np$ signify base fluid, nanofluid, and nanoparticles respectively.

Now, the similarity transformations are introduced as follow (see ref.^21^):

$$\eta =z\sqrt{\frac{{U}_{w}}{{x\nu }_{l}}}, u=ax{f}^{^{\prime}}\left(\eta \right), v=by{g}^{^{\prime}}\left(\eta \right), w=-\sqrt{{\nu }_{l}a}(f+g)$$

10 $$T=\left({T}_{f}-{T}_{\infty }\right)\theta \left(\eta \right)+{T}_{\infty }, C=\left({C}_{w}-{C}_{\infty }\right)\Theta \left(\eta \right)+{C}_{\infty }$$

here $\eta$ is the similarity variable, and $f, g,$ $\theta ,$ and $\Theta$ are respectively the dimensionless axial velocity, transverse velocity, temperature field, and nanoparticle volume fraction distributions of fields. Because of Eq.  (10), Eq. (1) satisfies trivially, and Eqs. (2)–(6) yields:

11 $$\frac{B}{A}{f}^{\prime\prime\prime}+\left(f+g\right){f}^{\prime\prime}-{{f}}^{{\prime\,{2}}}=0,$$

12 $$\frac{B}{A}{g}^{{\prime\prime\prime}}+\left(f+g\right){g}^{{\prime\prime}}-{{g}}^{{\prime\, {2} }}=0,$$

13 $$\frac{D}{Pr}{\theta }^{{\prime\prime}}+C(f+g){\theta }^{{\prime}}+CNb{\Theta }^{{\prime}}{\theta }^{{\prime}}+CNt{\theta }^{{{\prime}}2}+C{\beta }_{E}\mathrm{exp}(-n\eta )=0,$$

14 $${\Theta }{{^{\prime\prime}}}+\frac{Nt}{Nb}{\theta }{{^{\prime \prime}}}+Le(f+g){\Theta }{^{\prime}}=0,$$

With boundary conditions

15 $$\left.\begin{array}{c}f=g=0, {f}^{{\prime}}=1, {g}^{{\prime}}=\lambda , {\theta }{^{\prime}}=\frac{Bi\left(\theta -1\right)}{D}, \Theta =1 at \eta =0 \\ \\ {f}{^{\prime}}=0, {g}{^{\prime}}=0, \theta =0, \Theta =0 as \eta \to \infty . \end{array}\right\}$$

where,

$$A=\left(1-\phi \right)+\phi \frac{{\rho }_{np}}{{\rho }_{l}},$$

$$B=\frac{1}{{\left(1-\phi \right)}^{2.5}},$$

$$C=\left(1-\phi \right)+\phi (\rho {{C}_{p})}_{np }/(\rho {{C}_{p})}_{l},$$

$$D=\frac{{k}_{np}+2{k}_{l}-2\phi \left({k}_{l}-{k}_{np}\right)}{{k}_{np}+2{k}_{l}+\phi \left({k}_{l}-{k}_{np}\right)},$$

$\mathit{Pr}=\frac{{\left({C}_{p}\mu \right)}_{l}}{{k}_{l}}$ is the Prandtl number, $\lambda =\frac{b}{a}$ is the stretching ratio parameter, $Le=\frac{{\nu }_{l}}{{D}_{B}}$ is the Lewis number, $Nt=\frac{{\left(\rho {C}_{p}\right)}_{np}{D}_{T}\left({T}_{f}-{T}_{\infty }\right)}{{\left(\rho {C}_{p}\right)}_{l}{T}_{\infty }{\nu }_{l}}$ is the thermophoresis parameter, $Nb=\frac{{\left(\rho {C}_{p}\right)}_{np}{D}_{B}\left({C}_{w}-{C}_{\infty }\right) }{{\left(\rho {C}_{p}\right)}_{l}{\nu }_{l}}$ is the Brownian motion parameter, ${\beta }_{E}=\frac{{\beta }_{e}}{{\left(\rho {C}_{p}\right)}_{l}a}$ is the Exponential space-related heat source parameter (ESHS parameter) and $Bi=\frac{{h}_{l}}{{k}_{l}}\sqrt{\frac{{\nu }_{l}}{c}}$ is the Biot number.

The non-dimensional forms of friction factors along *x-* and *y-*directions ${Sf}_{x} \& {Sf}_{y}$, local Nusselt number $N{u}_{x}$ and local Sherwood number $S{h}_{x}$ are given by:

$$R{e}_{x}^{0.5}{Sf}_{x}=Bf{^{\prime\prime}}\left(0\right),$$

$$R{e}_{y}^{0.5}{Sf}_{y}=Bg{^{\prime\prime}}\left(0\right),$$

$$R{e}_{x}^{-0.5}N{u}_{x}=-D{\theta }{^{\prime}}\left(0\right)$$

16 $$R{e}_{x}^{-0.5}S{h}_{x}=-{\Theta }{^{\prime}}\left(0\right)$$

where $R{e}_{x}=\frac{{U}_{w}(x)x}{{\nu }_{l}}$ and $R{e}_{y}=\frac{{V}_{w}(y)y}{{\nu }_{l}}$ are local Reynolds numbers.

## Numerical method and validation

The nonlinear problem presented in the Eqs. (11)–(15) is numerically solved using the Finite Difference Method (FDM). The following substitutions are used $f={y}_{1}, {f}{^{\prime}}={y}_{2}, {f}{{^{\prime\prime}}}={y}_{3}, g={y}_{4}, {g}{^{\prime}}={y}_{5}, { g}{{^{\prime\prime}}}={y}_{6},\theta ={y}_{7}$, ${\theta }{^{\prime}}={y}_{8}$, $\Theta ={y}_{9}$ and ${\Theta }{^{\prime}}={y}_{10}$ to get the following :

17 $${y}_{1}{^{\prime}}={y}_{2,}$$

18 $${y}_{2}{^{\prime}}={y}_{3,}$$

19 $${y}_{3}{^{\prime}}=-\frac{A}{B}\left({y}_{1}+{y}_{4}\right){y}_{3}+\frac{A}{B}{\left({y}_{2}\right)}^{2},$$

20 $${y}_{4}{^{\prime}}={y}_{5,}$$

21 $${y}_{5}{^{\prime}}={y}_{6,}$$

22 $${y}_{6}{^{\prime}}=-\frac{A}{B}\left({y}_{1}+{y}_{4}\right){y}_{6}+\frac{A}{B}{\left({y}_{5}\right)}^{2},$$

23 $${y}_{7}{^{\prime}}={y}_{8},$$

24 $${y}_{8}{^{\prime}}=\frac{-\mathit{Pr}\left\{C\left({y}_{1}+{y}_{4}\right){y}_{8}+CNb{y}_{8}{y}_{10}+CNt{\left({y}_{8}\right)}^{2}+C{\beta }_{E}\mathrm{exp}\left(-n\eta \right)\right\}}{D},$$

25 $${y}_{9}{^{\prime}}={y}_{10},$$

26 $${y}_{10}{^{\prime}}=-LePr({y}_{1}+{y}_{4}){y}_{10}-\left(\frac{Nt}{Nb}\right){y}_{8}{^{\prime}},$$

with

$${y}_{1}\left(0\right)=0, {y}_{4}\left(0\right)=0, {y}_{2}\left(0\right)=1, {y}_{5}\left(0\right)=\lambda , {y}_{9}\left(0\right)=1, {y}_{8}\left(0\right)=\frac{Bi}{D}({y}_{7}\left(0\right)-1)$$

27 $${y}_{2}\left(\infty \right)=0, {y}_{5}\left(\infty \right)=0, {y}_{7}\left(\infty \right)=0, {y}_{9}\left(\infty \right)=0$$

The first-order system is solved via the bvp5c routine of MATLAB (see Ref.^44^). The bvp5c technique integrates a system set of differential equations of the form *y′* = *f(x,y)*, subject to the boundary conditions. bvp5c routine uses FDM with an achievable accuracy of about 10^–8^. Having condition at infinity is rescaled to 5. The obtained numeric data *–θ'(0)* is compared with the published studies when $Nb=Nt=\lambda ={\beta }_{E}=\phi =0$ and $Bi=\mathrm{10,000}$ for method’s validation. It is clear from Table 1 that the current study results are in good agreement with the results from the literature. In the next section, a parametric analysis is performed.

**Table 1:** Comparison of *–θ'(0)* values with those of Khan and Pop^24^ and Gorla and Sidawi^26^ when $Nb=Nt=\lambda ={\beta }_{E}=\phi =0$ and $Bi=\mathrm{10,000}$.

$P{r}_{l}$	Khan and Pop^24^	Gorla and Sidawi^26^	Present (bvp5c)
0.07	0.0663	0.0656	0.06562
0.2	0.1691	0.1691	0.16909
0.7	0.4539	0.5349	0.45392
2	0.9113	0.9114	0.91136
7	1.8954	1.8905	1.89542

## Results and discussion

**Figure 2:**
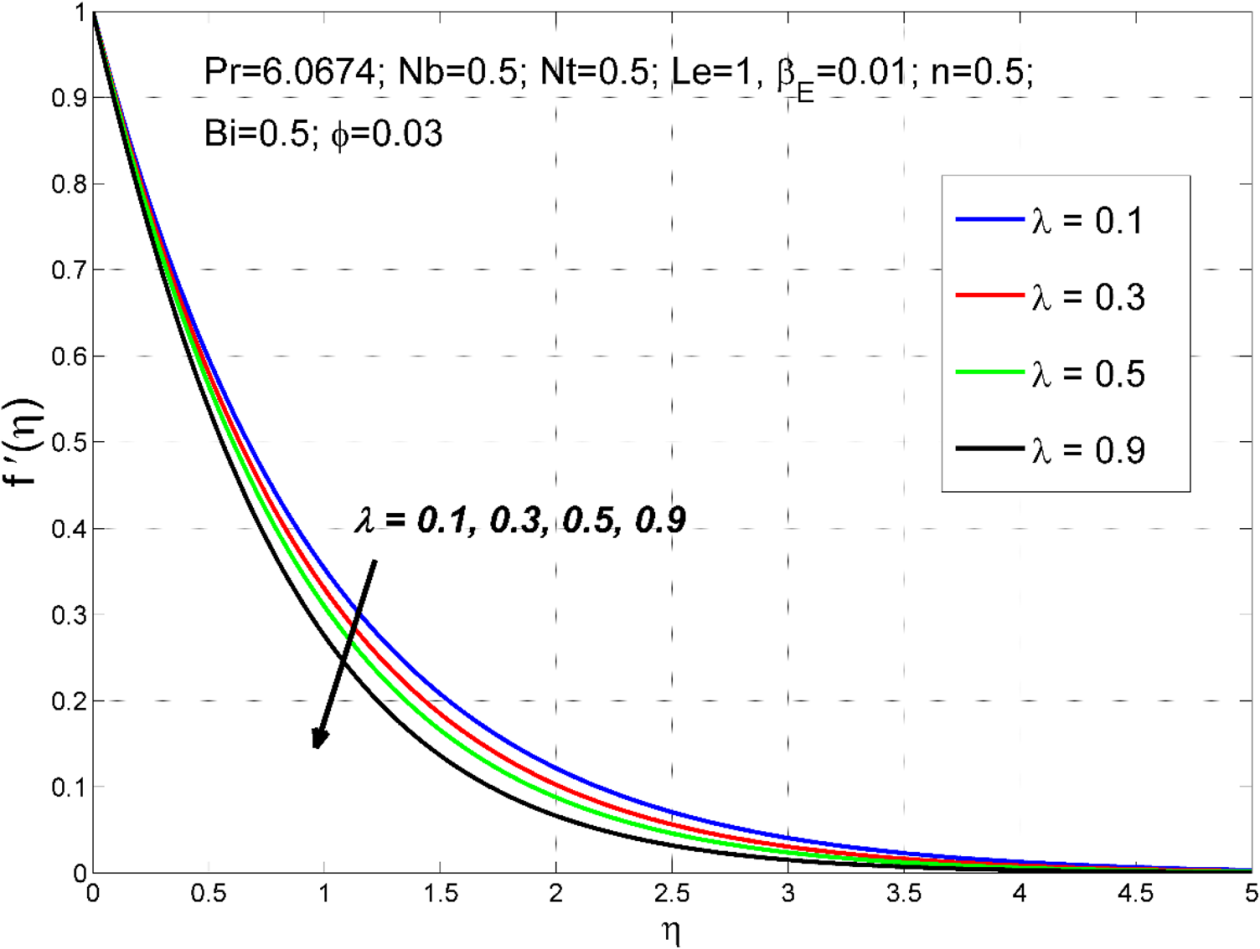
The axial velocity $f^{{\prime}}(\eta )$ behavior for the variation of the stretching ratio $\lambda$.

**Figure 3:**
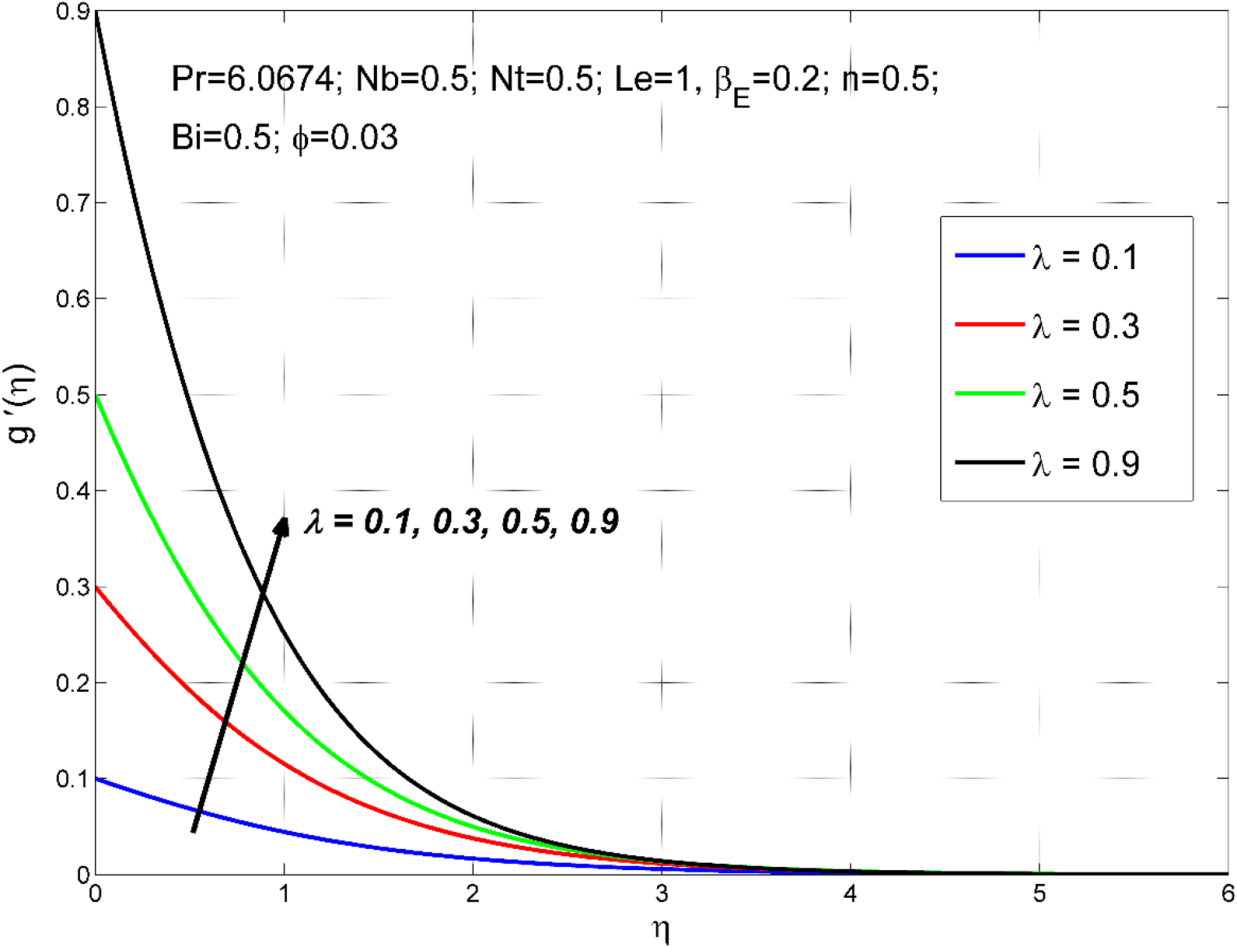
The transverse velocity $g{^{\prime}}(\eta )$ behavior for the variation of the stretching ratio $\lambda$.

**Figure 4:**
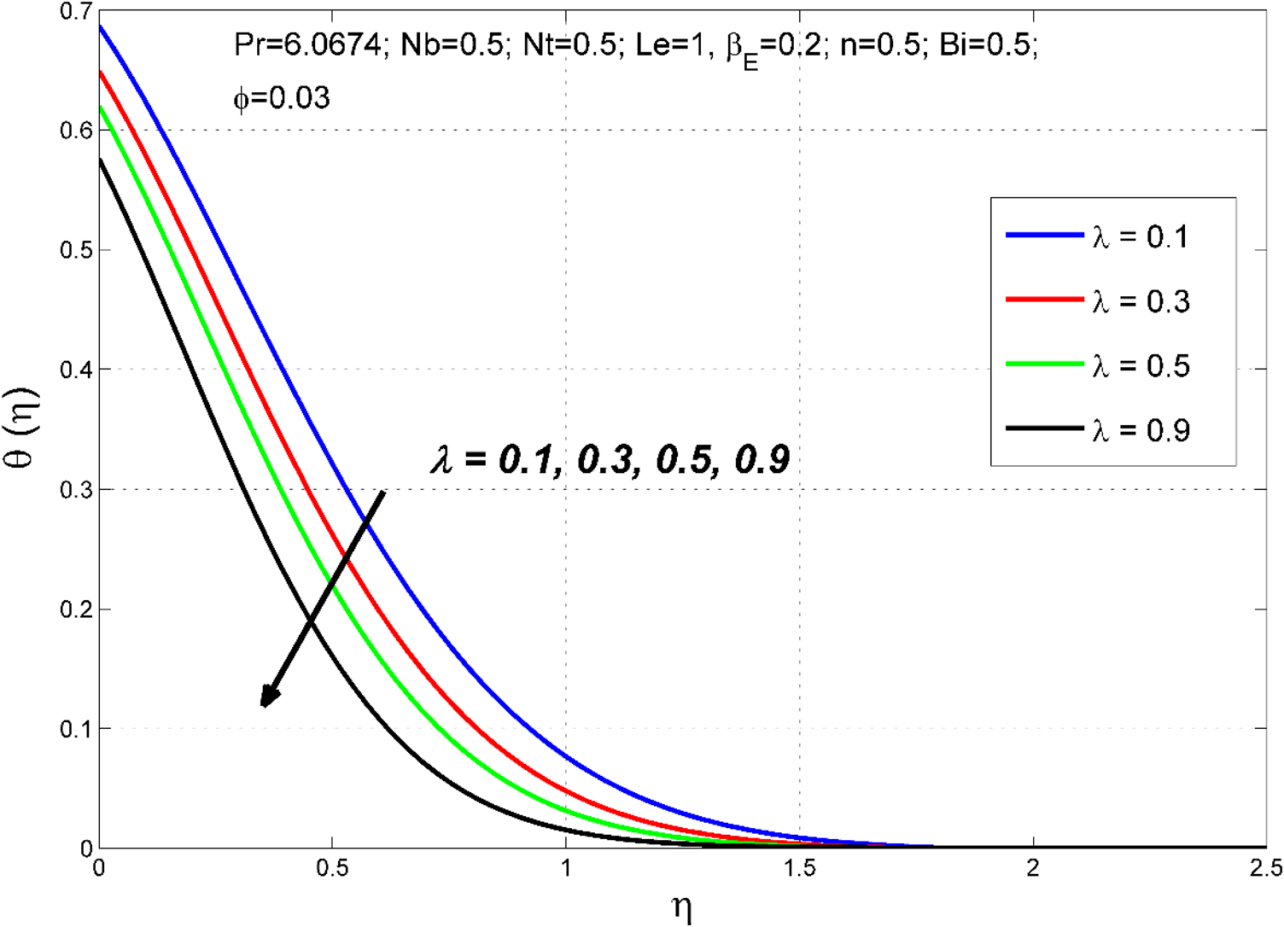
The temperature distribution $\theta (\eta )$ behavior for the variation of the stretching ratio $\lambda$.

**Figure 5:**
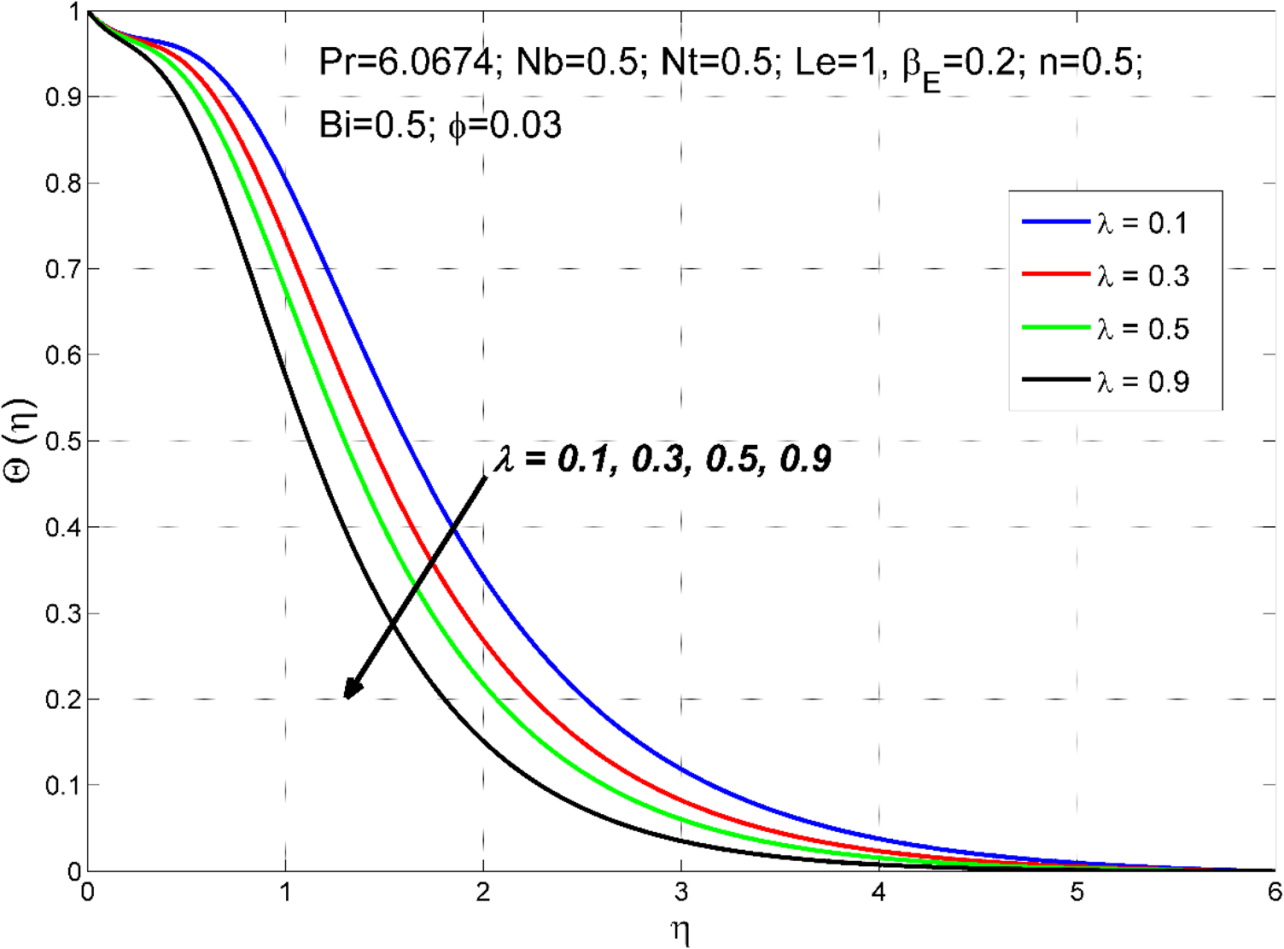
The nanoparticles volume fraction distribution $\Theta (\eta )$ behavior for the variation of the stretching ratio $\lambda$.

**Figure 6:**
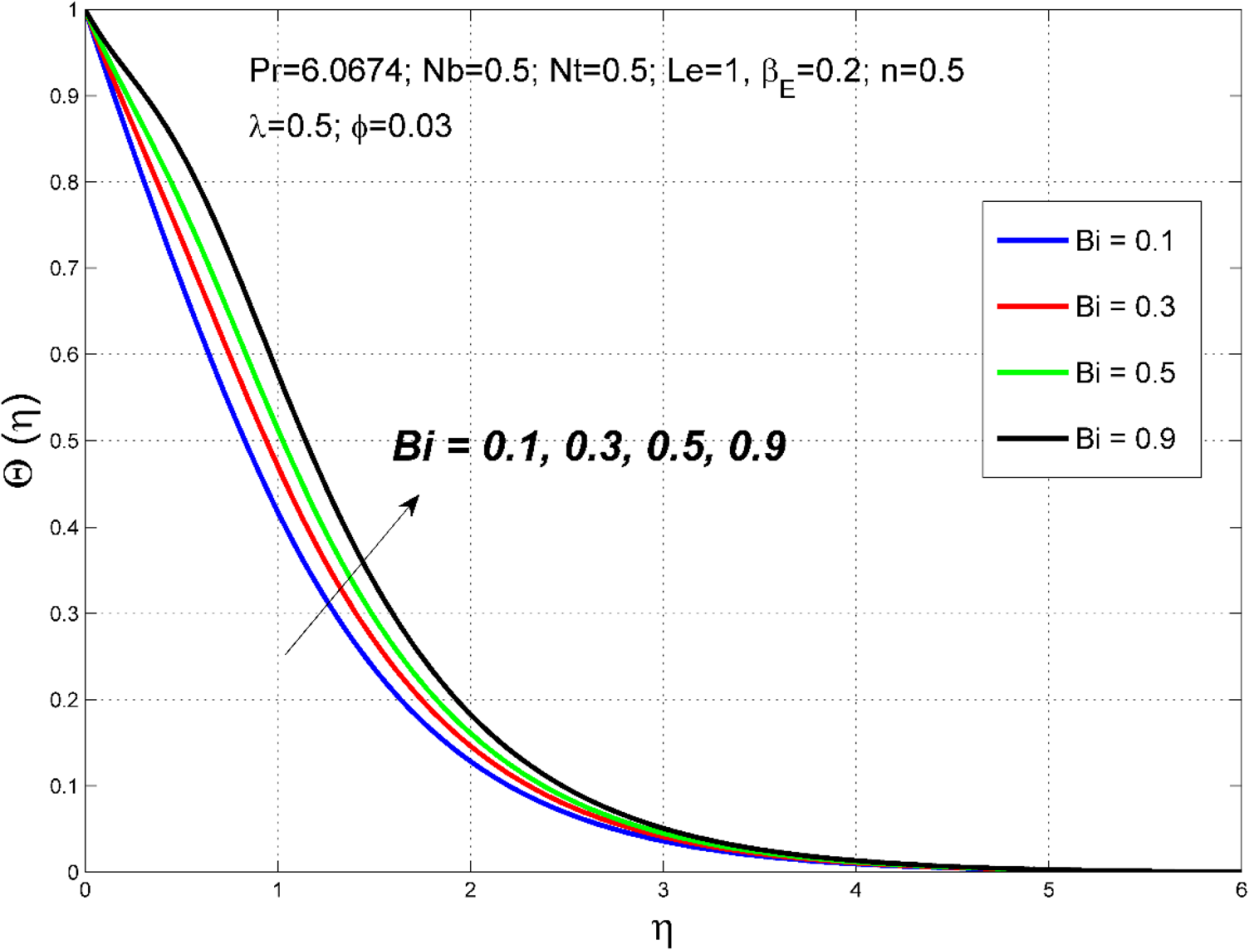
The nanoparticles volume fraction distribution $\Theta (\eta )$ behavior for the variation of the Biot number $Bi$.

**Figure 7:**
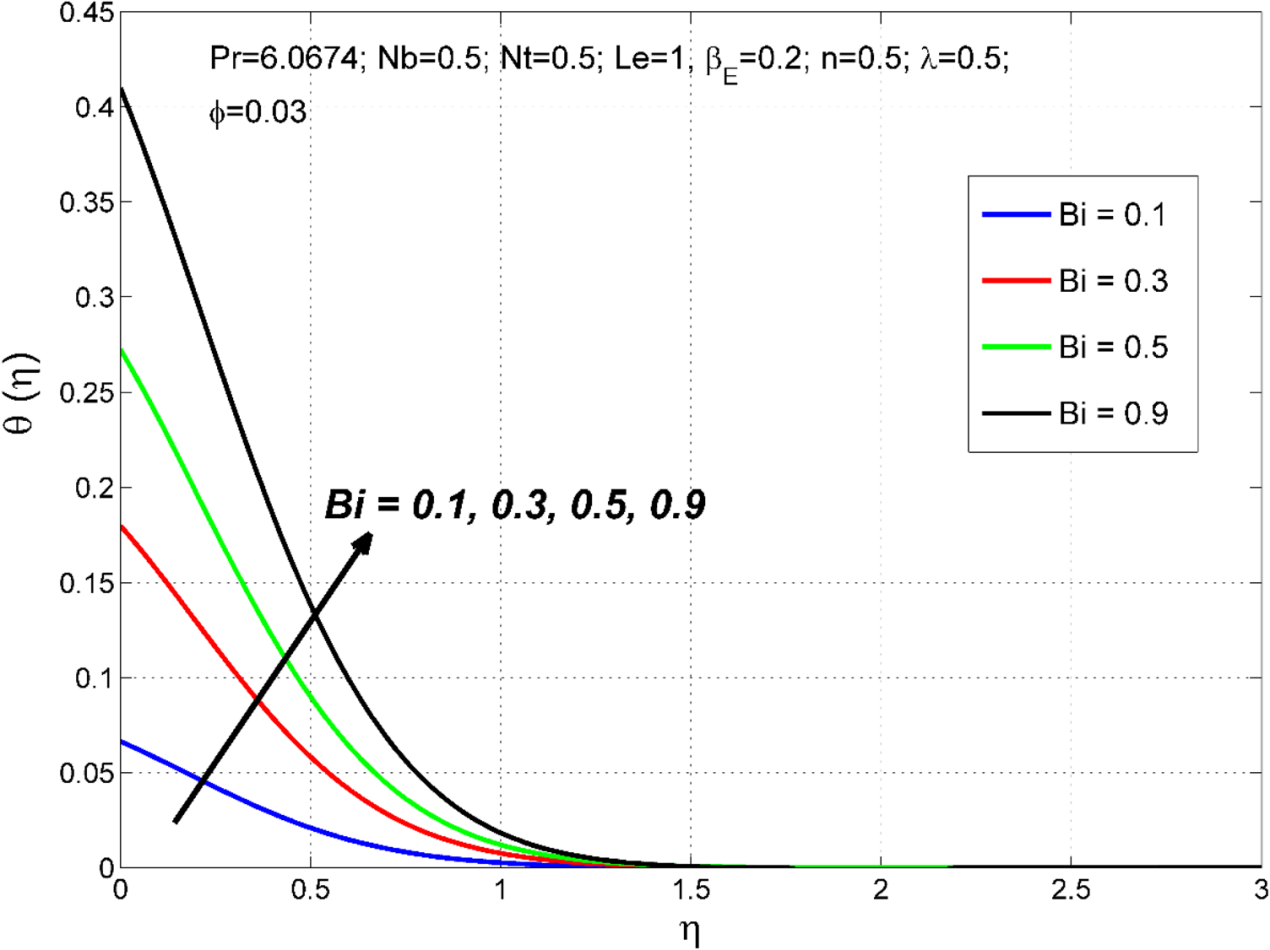
The temperature distribution $\uptheta (\eta )$ behavior for the variation of the Biot number $Bi$.

**Figure 8:**
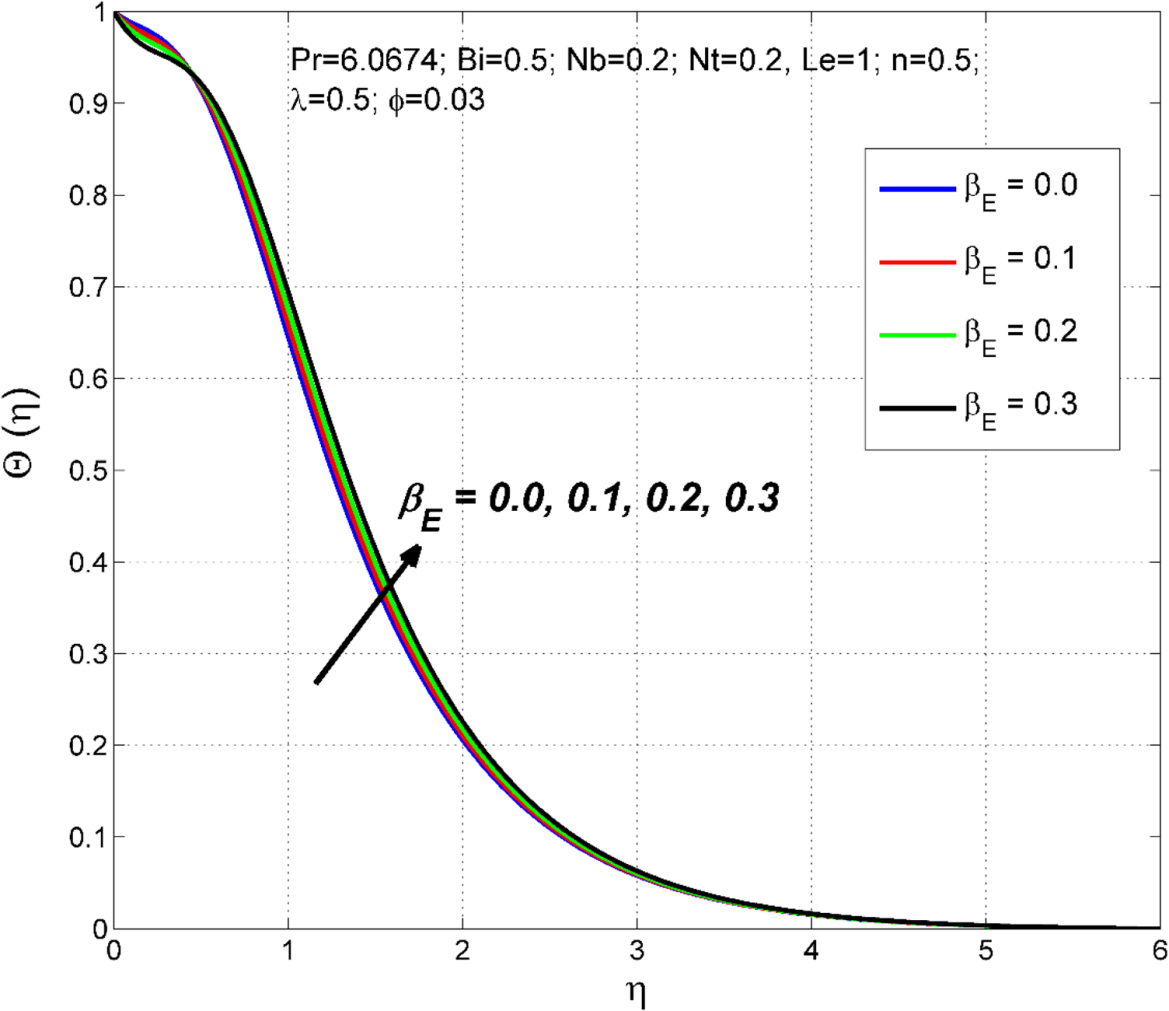
The nanoparticles volume fraction distribution $\Theta (\eta )$ behavior for the variation of the exponential heat source ${\beta }_{E}$.

**Figure 9:**
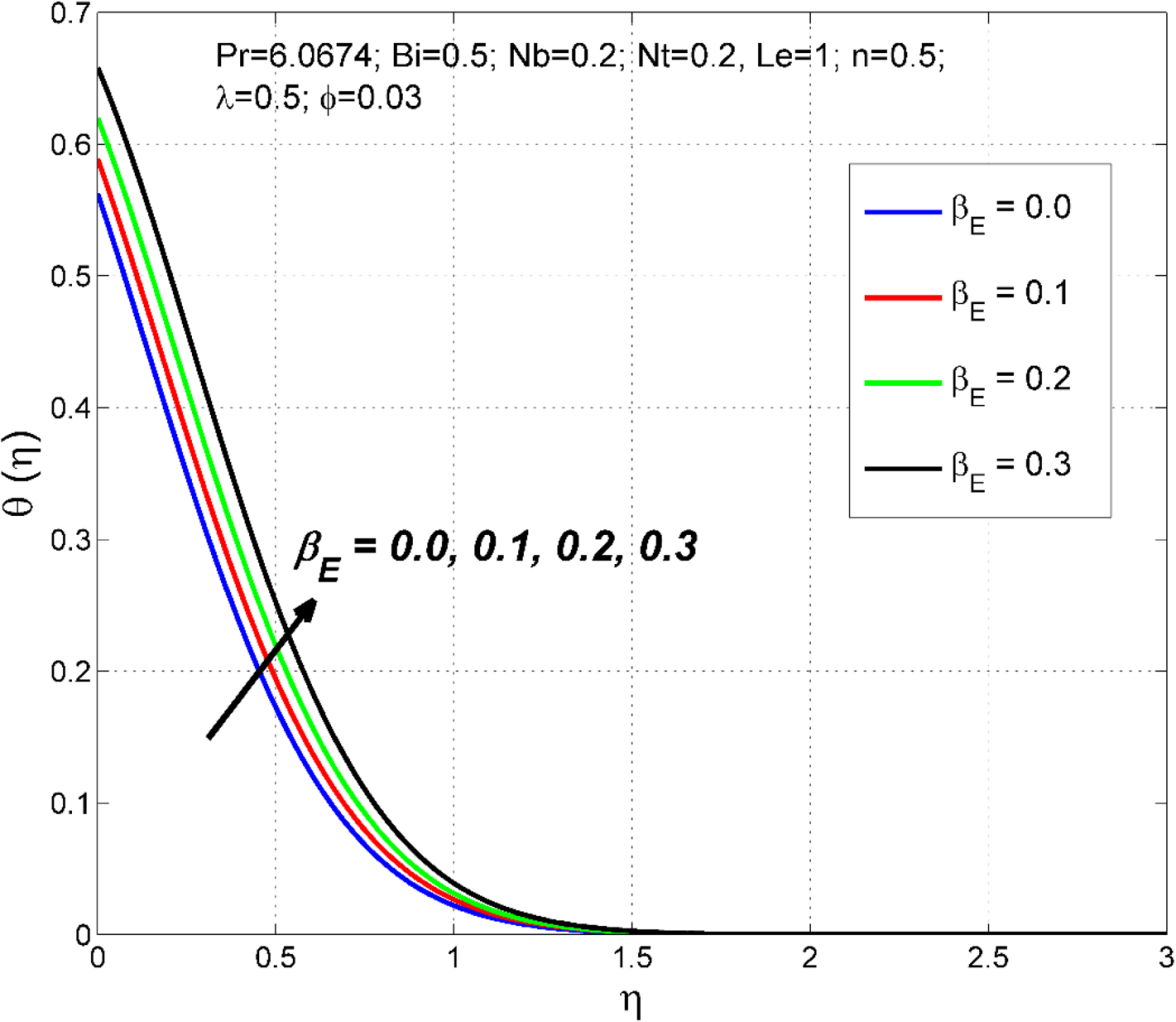
The temperature distribution $\uptheta (\eta )$ behavior for the variation of the exponential heat source ${\beta }_{E}$.

**Figure 10:**
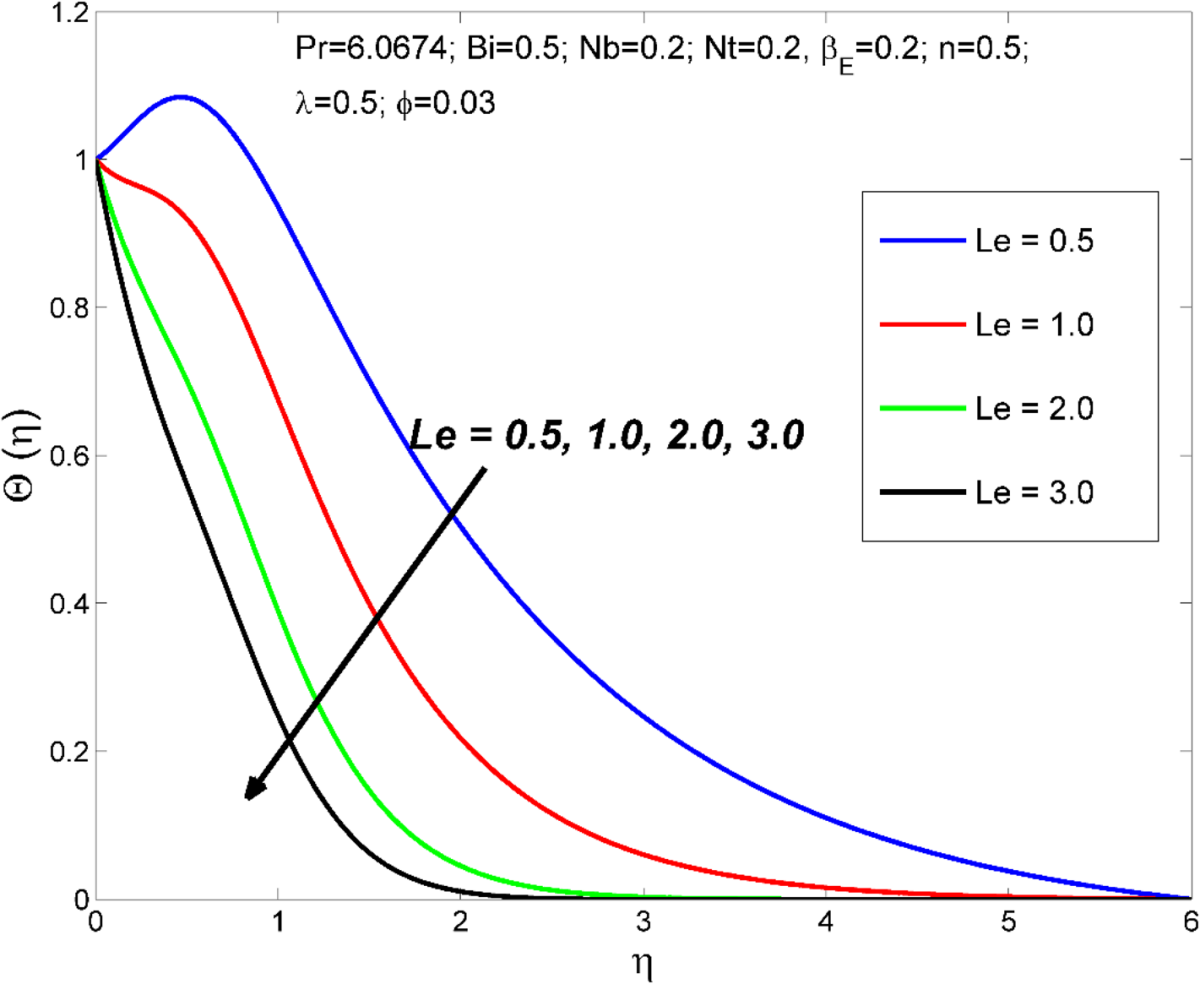
The nanoparticles volume fraction distribution $\Theta (\eta )$ behavior for the variation of the Lewis number $Le$.

**Figure 11:**
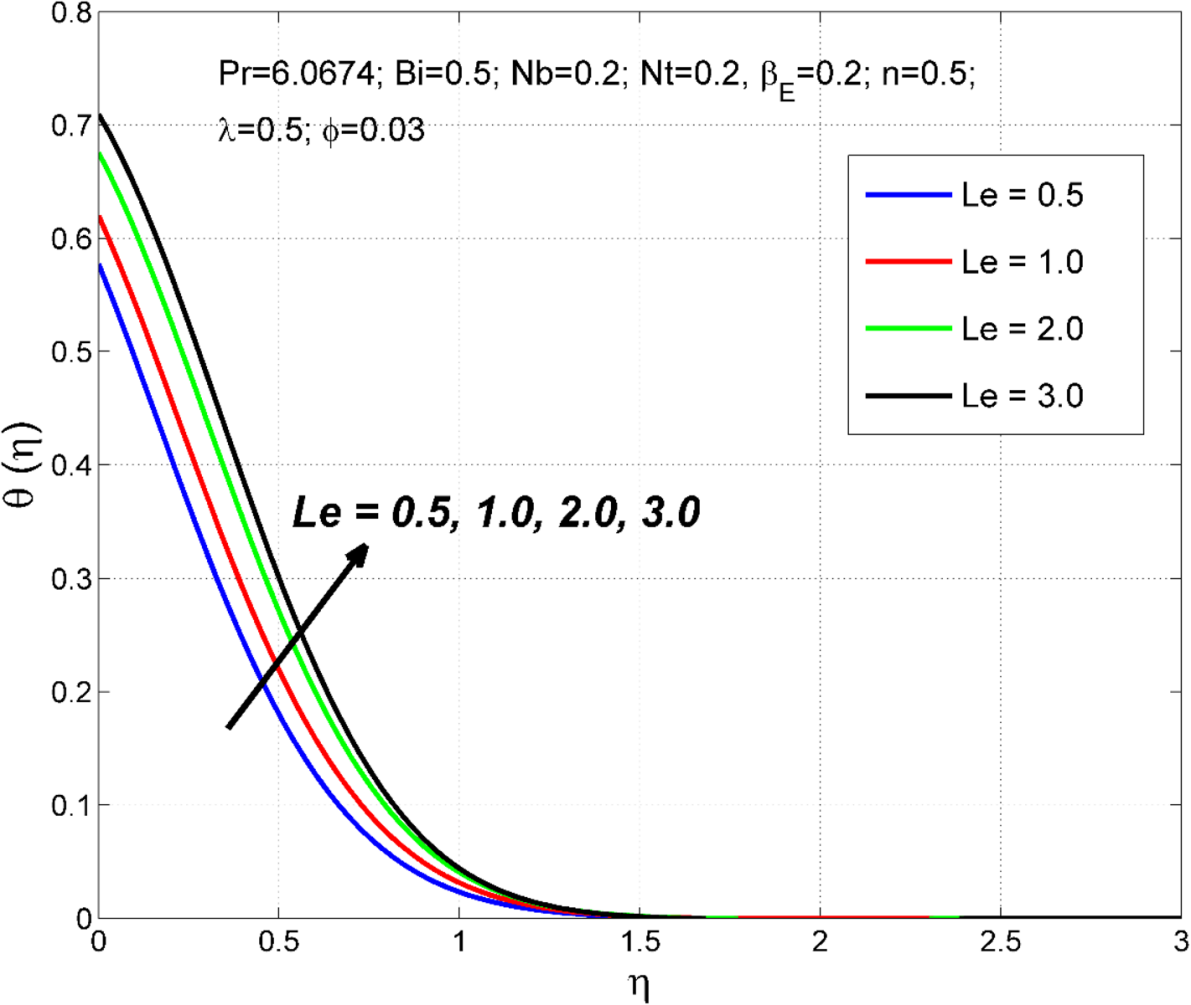
The temperature distribution $\uptheta (\eta )$ behavior for the variation of the Lewis number $Le$.

**Figure 12:**
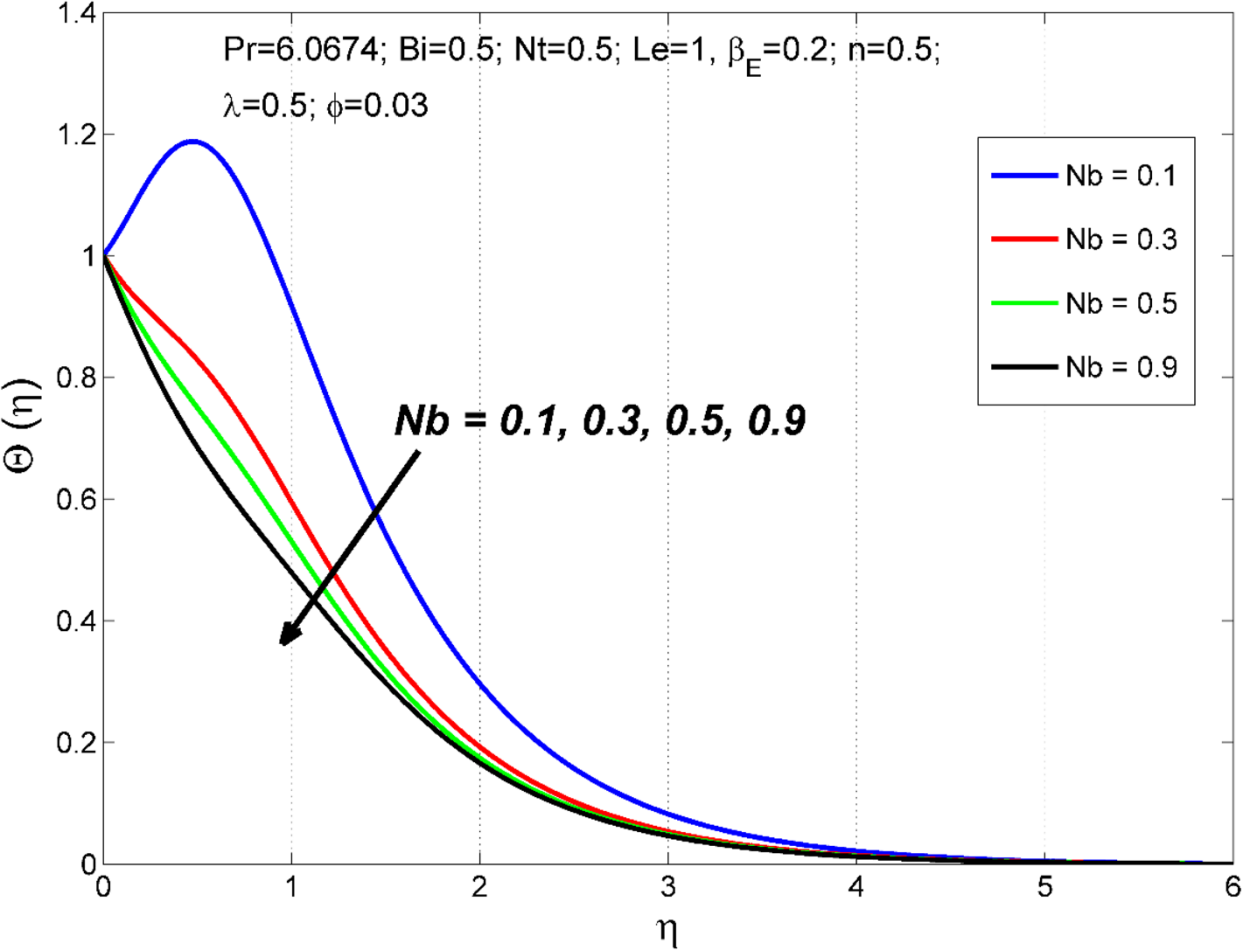
The nanoparticles volume fraction distribution $\Theta (\eta )$ behavior for the variation of the Brownian motion $Nb$.

**Figure 13:**
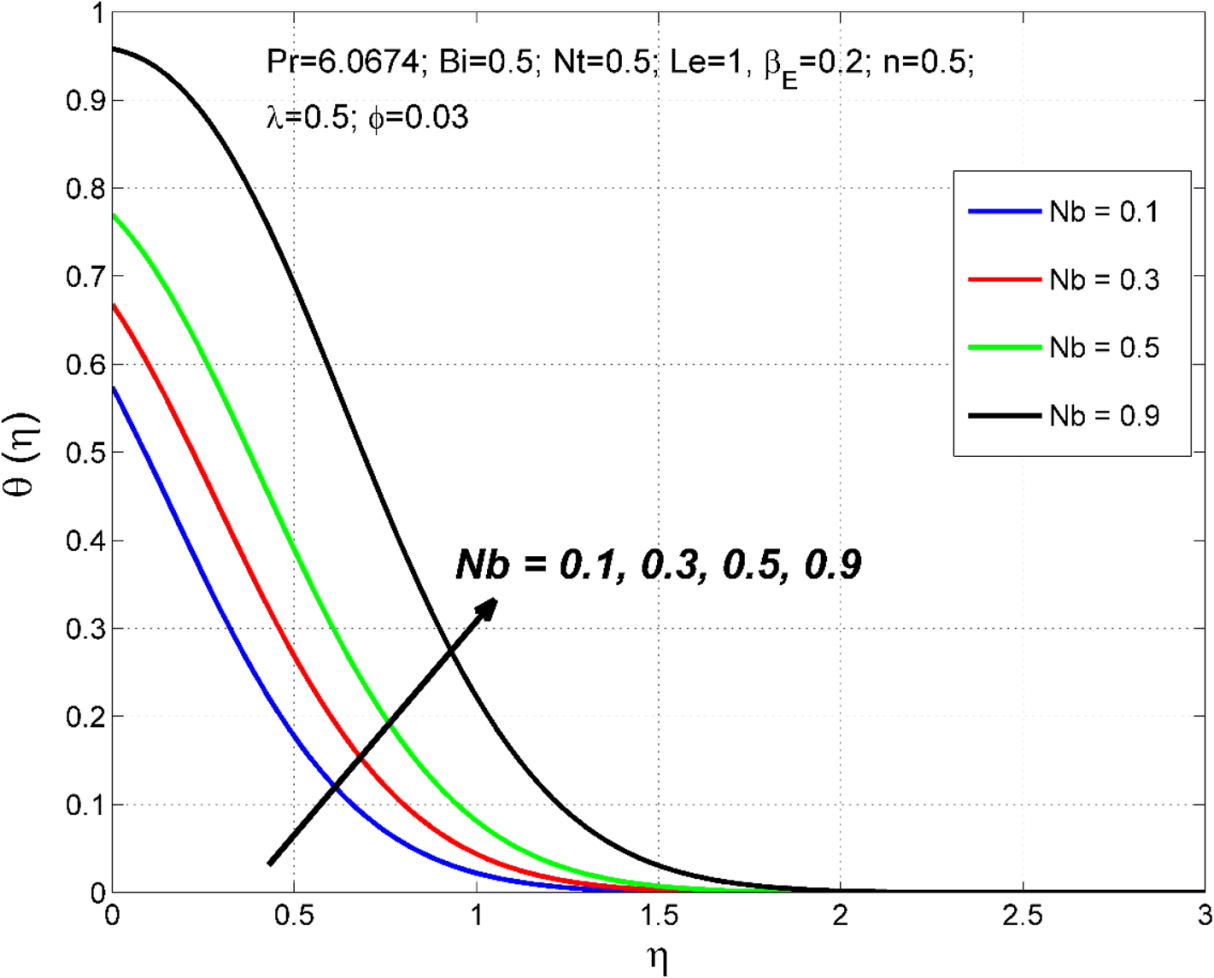
The temperature distribution $\uptheta (\eta )$ behavior for the variation of the Brownian motion $Nb$.

**Figure 14:**
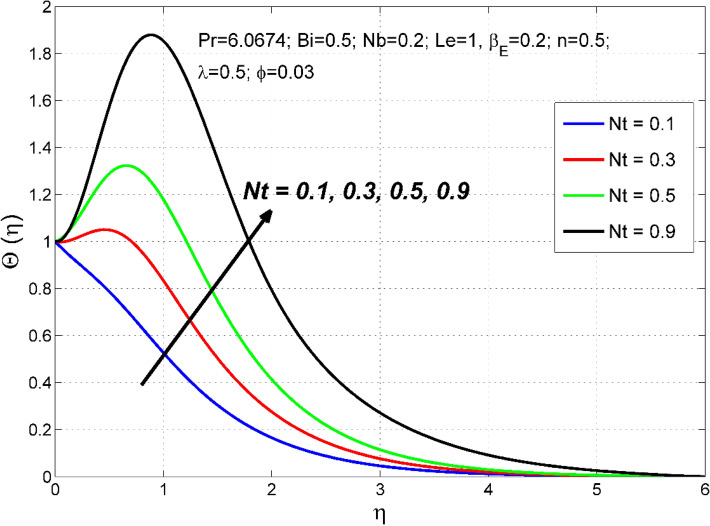
The nanoparticles volume fraction distribution $\Theta (\eta )$ behavior for the variation of the thermophoresis $Nt$.

**Figure 15:**
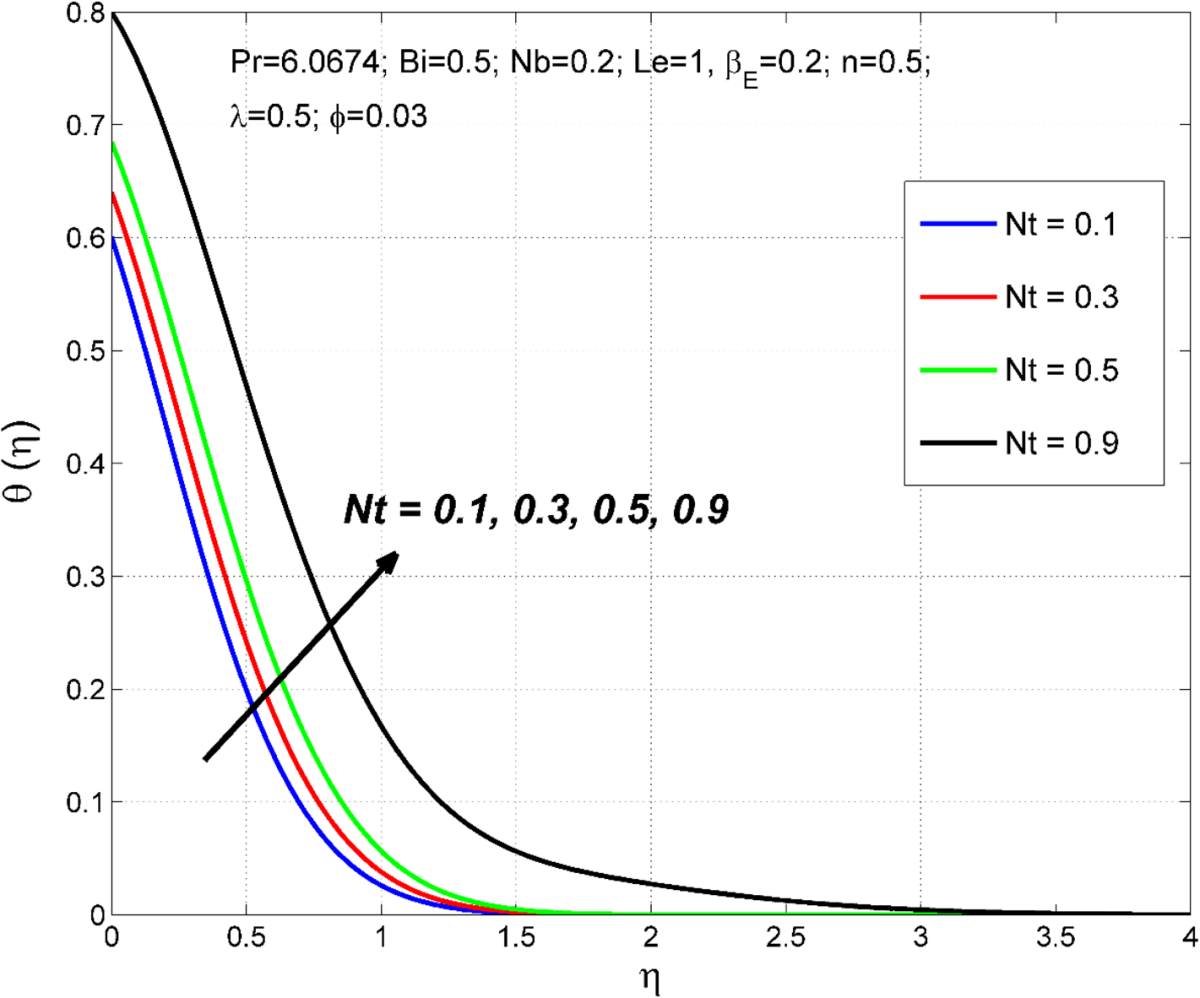
The temperature distribution $\uptheta (\eta )$ behavior for the variation of the thermophoresis $Nt$.

**Figure 16:**
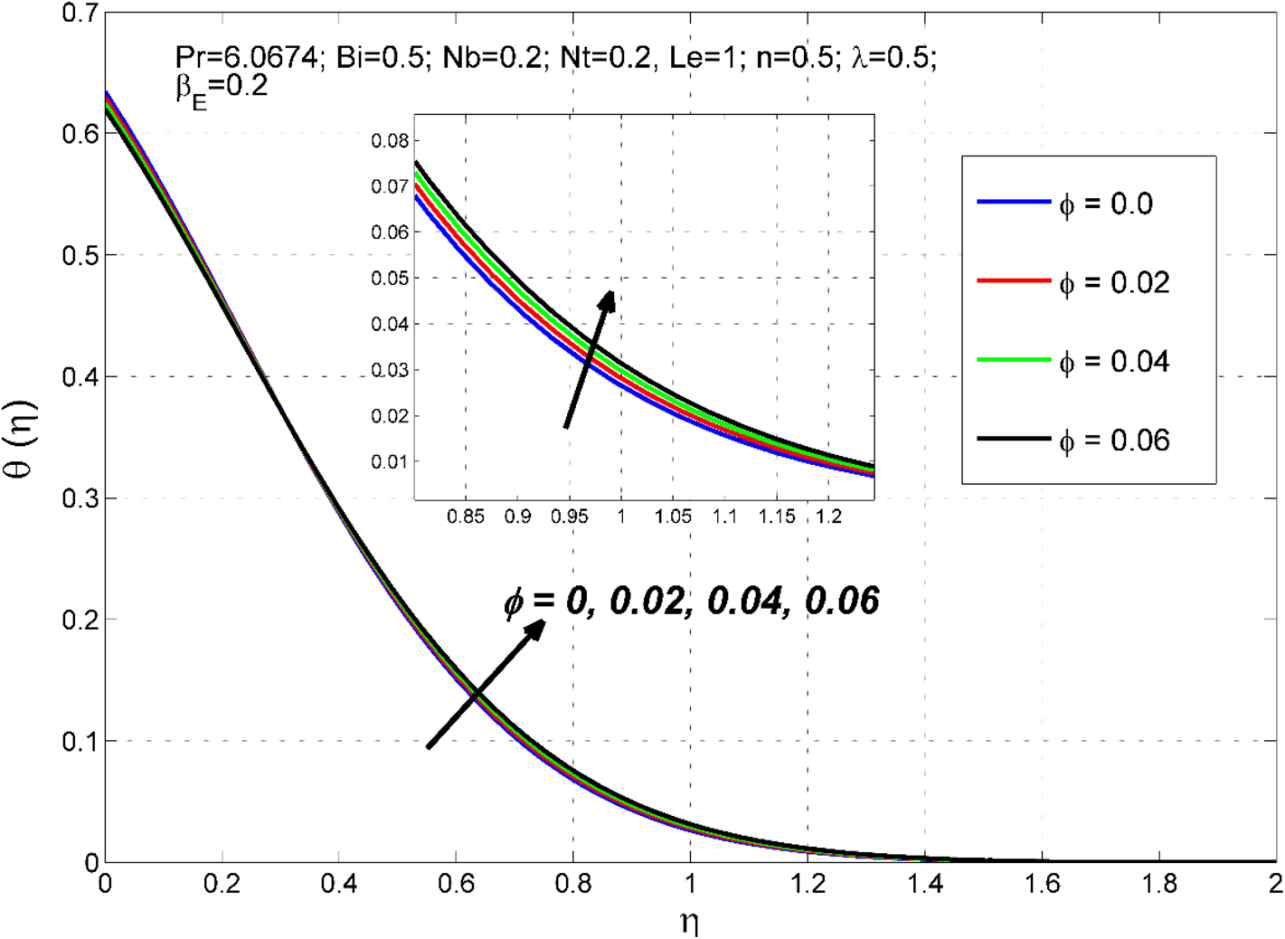
The temperature distribution $\uptheta (\eta )$ behavior for the variation of the nanoparticles volume fraction $\phi$.

**Figure 17:**
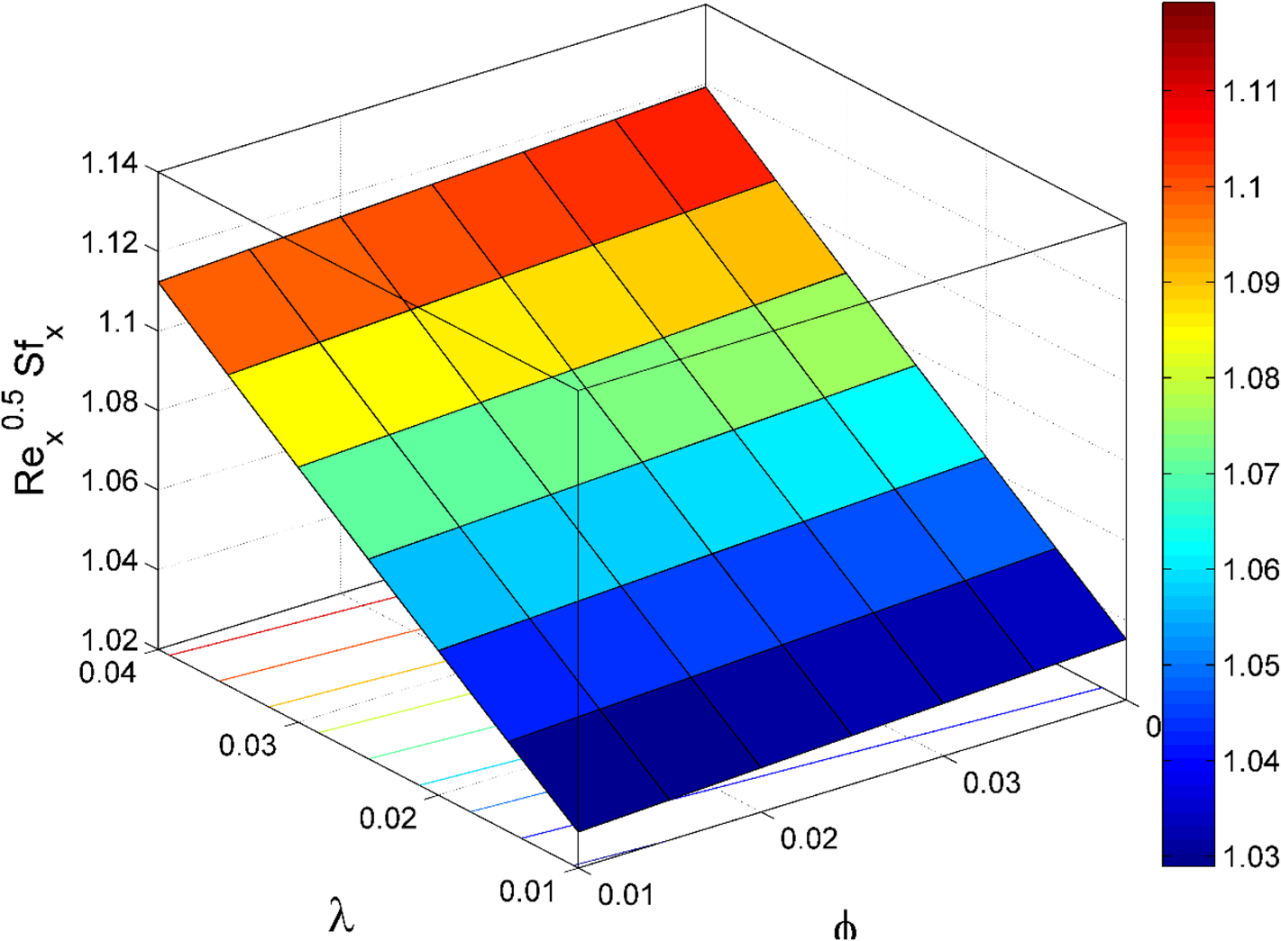
${\mathrm{Re}}_{\mathrm{x}}^{0.5}S{f}_{x}$ behavior for the variation of nanoparticles volume fraction $\phi$ and stretching ratio $\lambda$.

**Figure 18:**
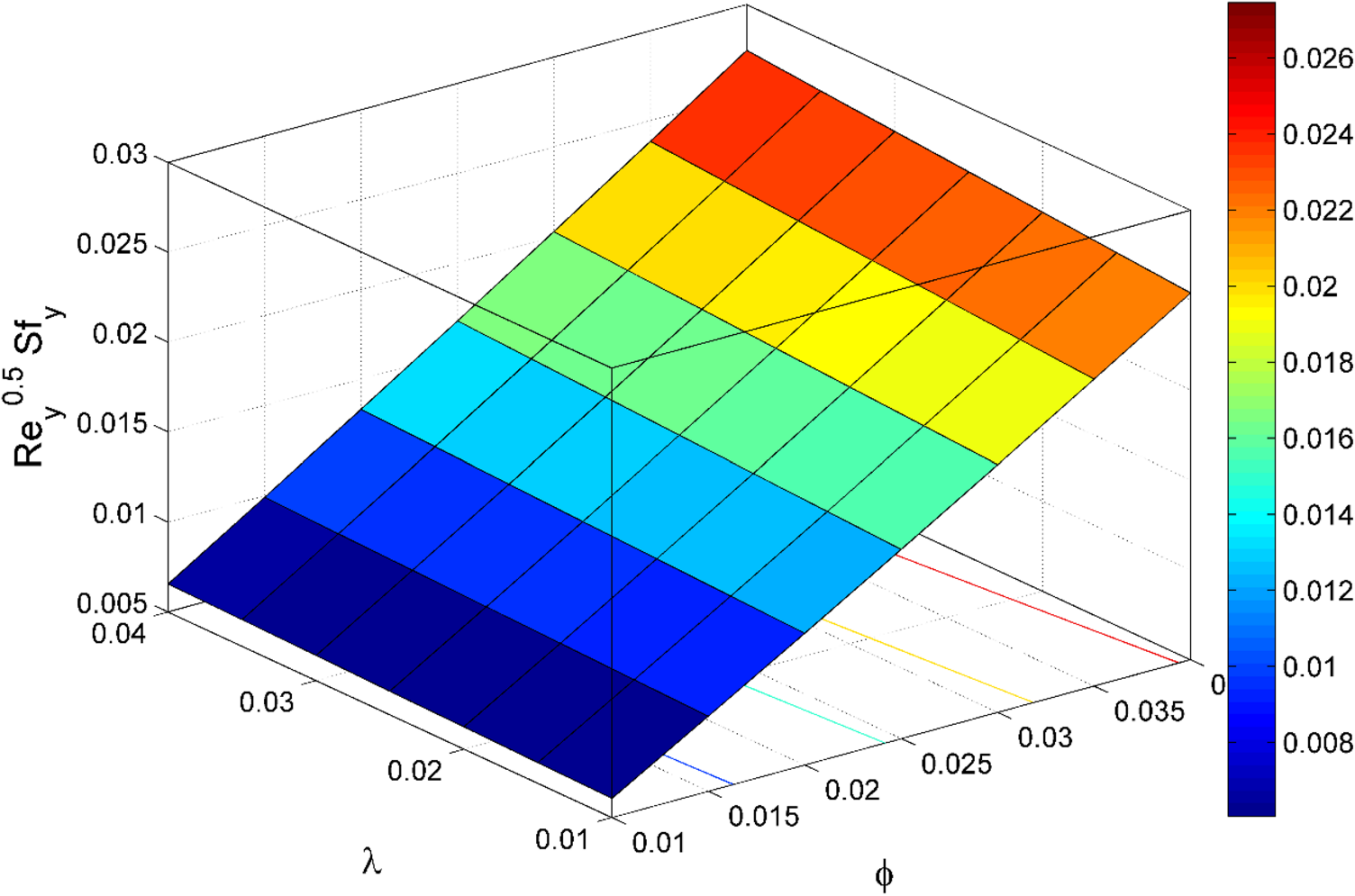
${\mathrm{Re}}_{\mathrm{y}}^{0.5}S{f}_{y}$ behavior for the variation of nanoparticles volume fraction $\phi$ and stretching ratio $\lambda$.

**Figure 19:**
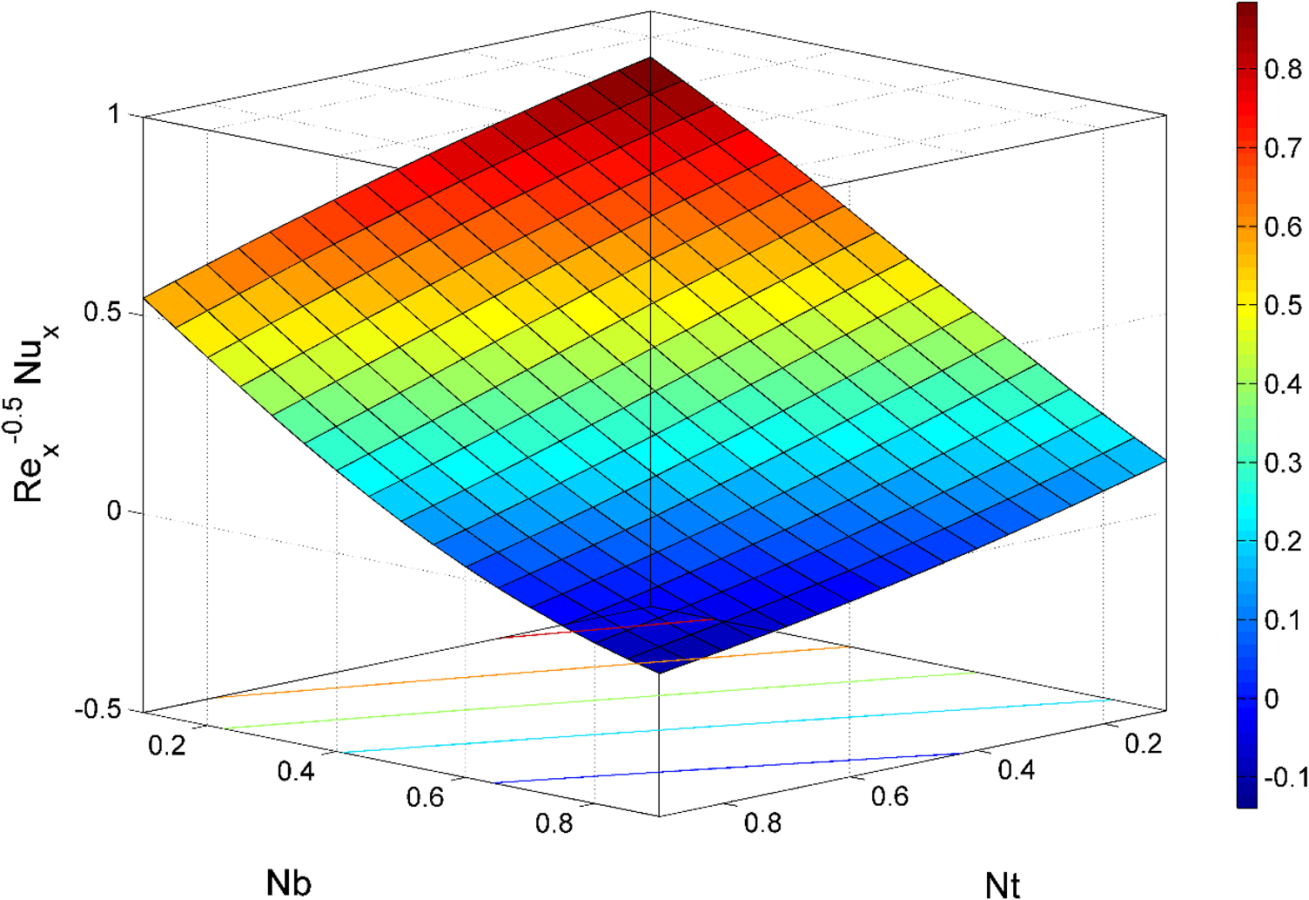
${\mathrm{Re}}_{\mathrm{y}}^{-0.5}N{u}_{x}$ behavior for the variation of Brownian motion $Nb$ and Thermophoresis $Nt$.

**Figure 20:**
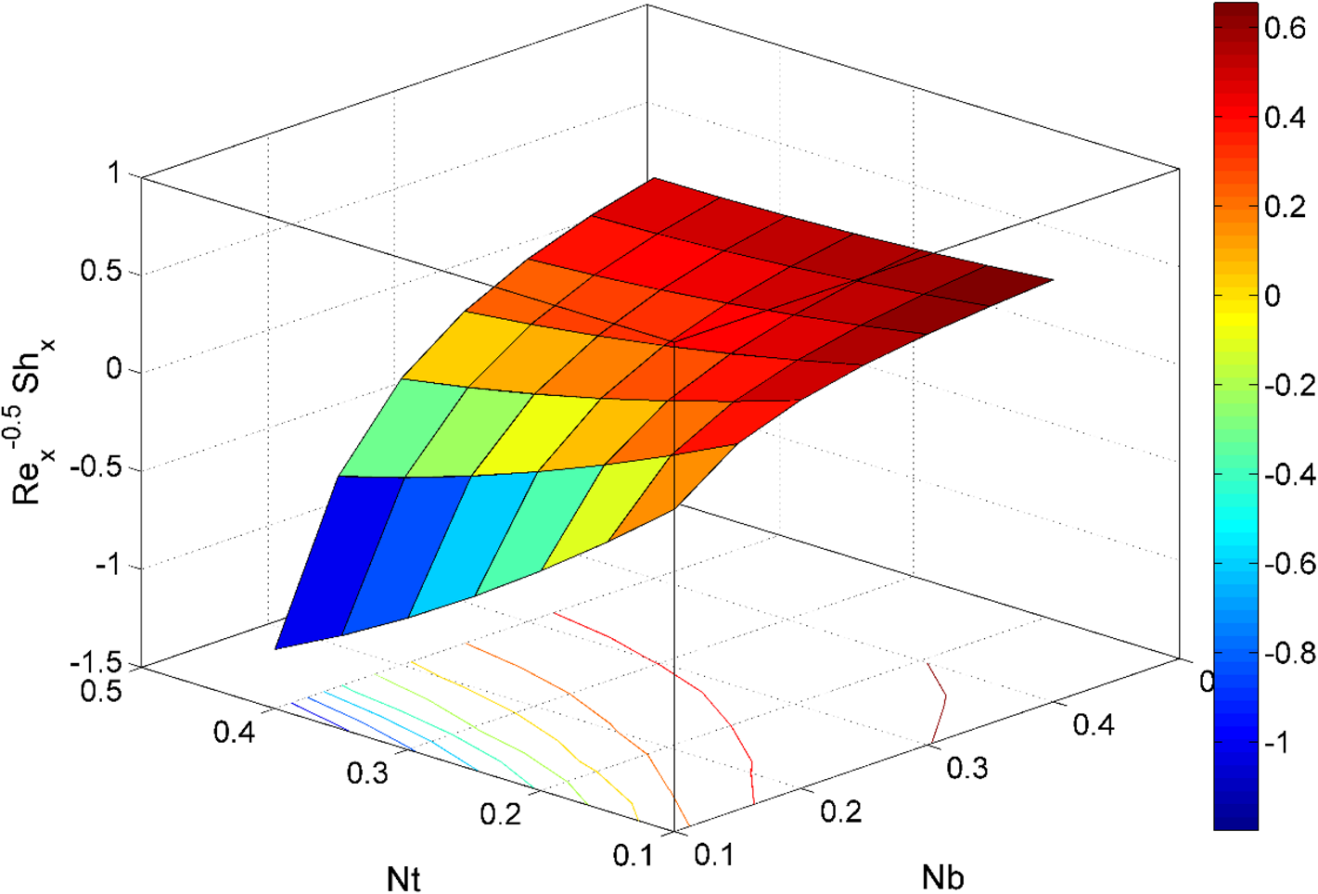
${\mathrm{Re}}_{\mathrm{y}}^{-0.5}S{h}_{x}$ behavior for the variation of Brownian motion $Nb$ and Thermophoresis $Nt$.

**Figure 21:**
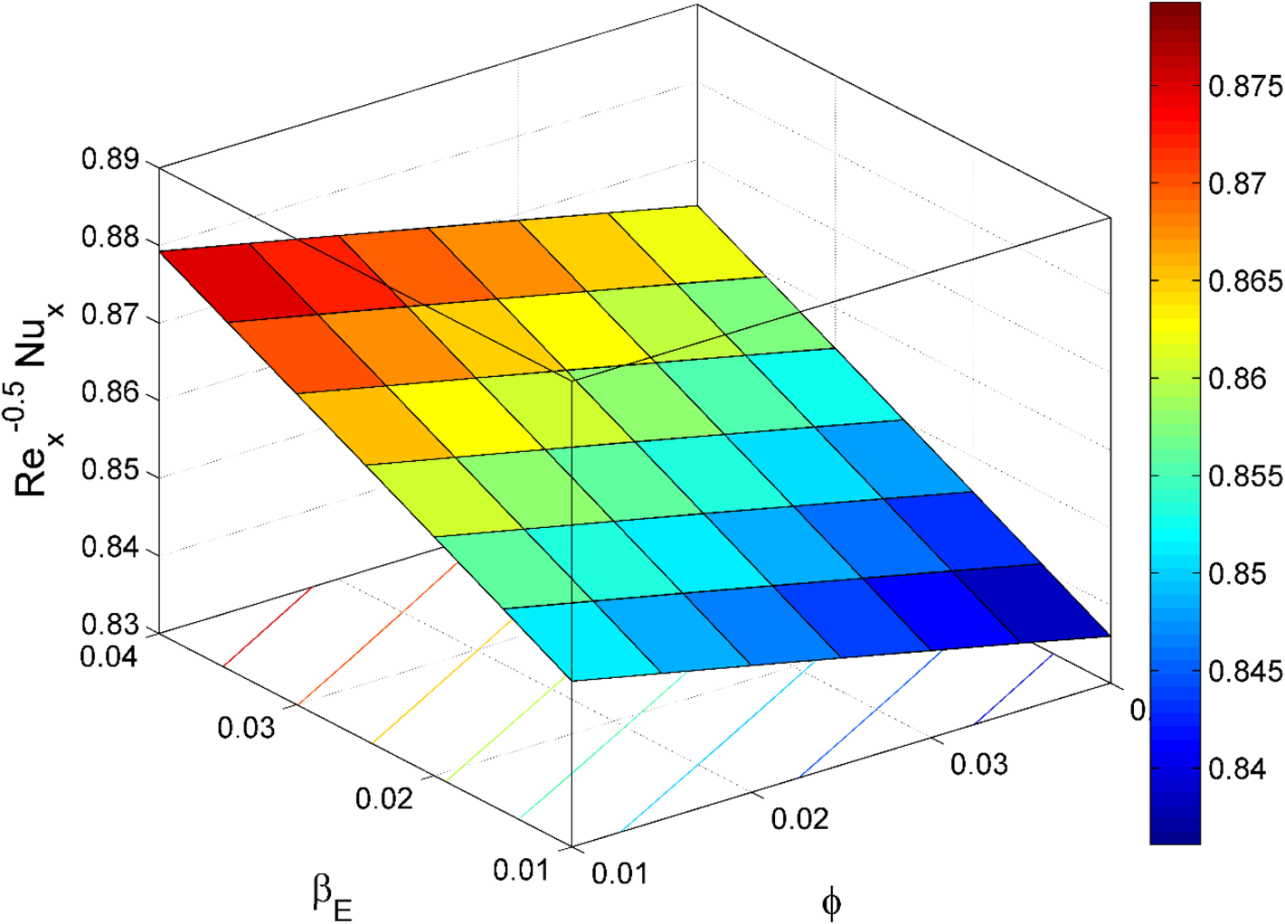
${\mathrm{Re}}_{\mathrm{y}}^{-0.5}N{u}_{x}$ behavior for the variation of heat source ${\beta }_{E}$ and nanoparticles volume fraction $\phi$.

**Figure 22:**
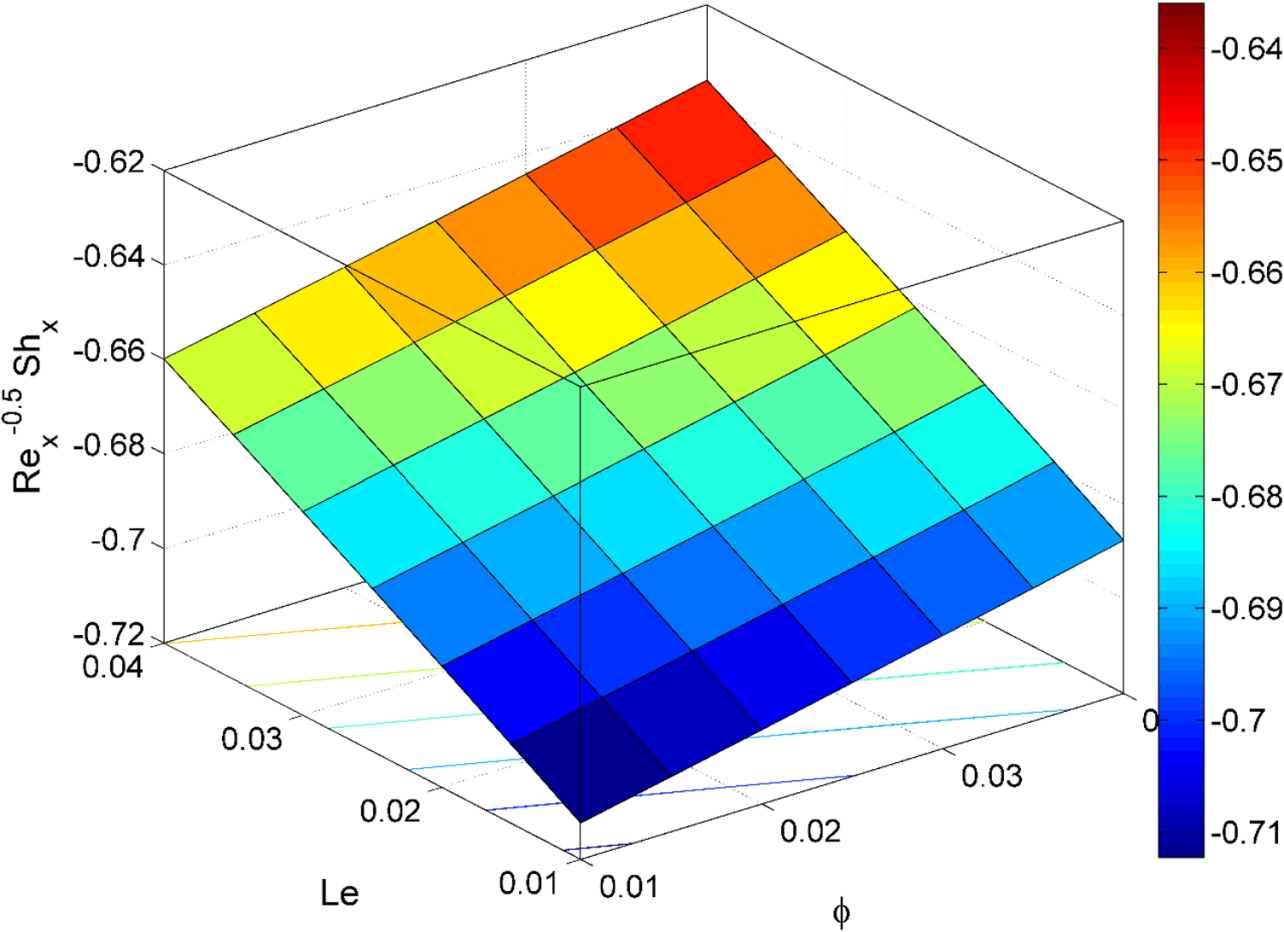
${\mathrm{Re}}_{\mathrm{y}}^{-0.5}S{h}_{x}$ behavior for the variation of Lewis number $Le$ and nanoparticles volume fraction $\phi$.

In this section, a comprehensive parametric study of the consequence of various governing dimensionless parameters is conducted. Parameters including stretching ratio parameter 0.1 $\le \lambda \le 0.9$, Biot number $0.1\le Bi\le 0.9$, exponential heat generation parameter $0{\le \beta }_{E}\le 0.3$, Lewis number $0.5\le Le\le 3.0$, Brownian motion parameter $0.1\le Nb\le 0.9$, thermo-migration parameter of $0.1\le Nt\le 0.9$ and copper nanoparticles volume fraction of $0\le \phi \le 0.06$ on velocities ${f}^{{\prime}}\left(\eta \right)\& g^{{\prime}}(\eta )$, temperature $\theta (\eta )$, dimensionless nanoparticle volume fraction $\Theta (\eta )$, wall friction coefficients $R{e}_{x}^{0.5}S{f}_{x} \& R{e}_{y}^{0.5}S{f}_{y}$, Sherwood number $R{e}_{x}^{-0.5}S{h}_{x}$ and $R{e}_{x}^{-0.5}N{u}_{x}$ Nusselt number fields. The results are presented in Figs. 2, 3, 4, 5, 6, 7, 8, 9, 10, 11, 12, 13, 14, 15, 16, 17, 18, 19, 20, 21 and 22 and the numeric values of the parameters are mentioned in the figures.

Figures 2, 3, 4 and 5 disclose the influence of the stretching ratio $\lambda =\frac{b}{a}$ on the behavior of velocities ${f}{^{\prime}}\left(\eta \right) \& g{^{\prime}}(\eta )$, temperature $\theta (\eta )$, and nanoparticle volume fraction $\Theta (\eta )$. The stretching ratio $\lambda$ enhanced either by increasing the starching rate $b$ along *y-*direction or decreasing the starching rate $a$ along the *x-*direction. Figures 2 and 3 indicating that the axial velocity ${f}{^{\prime}}\left(\eta \right)$ and interrelated layer width is condensed by enhancing the stretching ratio parameter ($\lambda$). However, the transverse velocity ${g}{^{\prime}}\left(\eta \right)\mathrm{}$ considerably enhanced by improving $\lambda$. This is because, the magnitude of the stretching velocity along with y-direction increases with $\lambda$, as a result, the $y$ component velocity increases with $\lambda$. Moreover, the thermal and nanoparticle concentration layer structure diminished with the values of $\lambda$ are increased. Moreover, Figs. 4 and 5 show a diminishing tendency of both the temperature $\theta (\eta )$ and nanoparticle volume fraction $\Theta (\eta )$ with declining $\lambda$.

Figures 6, 7 depict how the Biot number ($Bi$) affects the nanoparticle volume fraction $\Theta (\eta )$ and temperature $\theta (\eta )$ fields. Sturdier convective heat transfer at the film surface is formed by cumulative numerical values of $Bi$. Consequently, the thermal and nanoparticle concentration layers structure are increased.

The significance of ${\beta }_{E}$ on nanoparticle concentration $\Theta (\eta )$ and temperature $\theta (\eta )$ distributions are sketched in Figs. 8 and 9. ${\beta }_{E}$ is a quantity that measures the amount of internal heating in the nanofluid system. As ${\beta }_{E}$ increases, the amount of additional internal heat supplied to the nanofluid boundary layer increases, causing the nanofluid temperature to increase, hence the thermal boundary layer thickness. Thus, both nanoparticle concentration $\Theta (\eta )$ and temperature $\theta (\eta )$ distributions are improved.

Figures 10 and 11 disclose the influence of $Le$ on $\Theta \left(\eta \right)$ and $\theta (\eta )$. The effect of $Le$ on $\Theta \left(\eta \right)$ and $\theta (\eta )$ is qualitatively opposite. That is, the $\Theta \left(\eta \right)$ decreased and the $\theta (\eta )$ increased for enlarging numerical values of $Le$. The $Le$ is directly dependent on the Brownian diffusivity. A rise in $Le$ produces lower Brownian diffusivity and this is responsible for thinner thickness of nanoparticle volume fraction. Figures 12 and 13 reveal that the cause of $Le$ and $Nb$ on $\Theta \left(\eta \right)$ and $\theta \left(\eta \right)$ is qualitatively identical. That is, the magnitude of $\Theta \left(\eta \right)$ decreased and the magnitude of $\theta (\eta )$ increased for enlarging numerical values of $Nb$. Due to the nanoparticles' arbitrary movement and random collisions with the liquid base molecules, heat is produced in the nanofluid system. Thus, the magnitude of temperature distribution $\theta (\eta )$ increased along with its associated layer structure. Moreover, the nanoparticles' arbitrary movement decreases the nanoparticles volume fraction distribution $\Theta \left(\eta \right)$.

Thermophoresis parameter $Nt$ impact of on $\Theta \left(\eta \right)$ and $\theta (\eta )$ are demonstrated in Figs. 14 and 15 respectively. Thermophoresis is related to the movement of nanoparticles through the base fluid caused by the presence of a thermal gradient. A higher thermo-migration factor $Nt$ establishes developed temperature and nanoparticle volume fraction fields. This is because an augmentation in $Nt$ produces a sturdier thermophoretic force which consents to more profound migration of nanoparticles, as a result, the $\Theta \left(\eta \right)$ and $\theta (\eta )$ is increased with $Nt$.

The nanoparticles volume fraction $\phi$ importance on $\theta (\eta )$ is visualized in Fig. 16. It is observed that the $\theta (\eta )$ enhances with an increase in $\phi$ values. This is because of the superior thermophysical properties of the resulting nanofluid. Advancing the value of $\phi$ causes a higher thermal conductivity and thereby thermal field $\theta (\eta )$ of $Cu-{H}_{2}O$ nanofluid.

Figures 17 and 22 are drawn for the values of $Nb=Nt=0.2, Le=1, Pr= 6.0674, \lambda =0.5, Bi=2, {\beta }_{E}=0.2, n=0.01, \phi =0.03$ except when they are varied. The responses of wall friction coefficient along x and y directions ($R{e}_{x}^{0.5}S{f}_{x} \& R{e}_{y}^{0.5}S{f}_{y})$ for various values of $\phi$ and $\lambda$ are displayed in Figs. 17 and 18. Both $R{e}_{x}^{0.5}S{f}_{x}$ and $R{e}_{y}^{0.5}S{f}_{y}$ enhances rapidly by increasing $\phi$, this is because of thinner momentum layer thickness caused by higher volume fraction of nanoparticles. The local Nusselt number ${Re}_{\mathrm{x}}^{-0.5}N{u}_{x}$ for the distinction of $Nt$ and $Nb$ are illustrated in Figs. 19 and 20. It is perceived that the ${Re}_{\mathrm{x}}^{-0.5}N{u}_{x}$ is a decreasing property of $Nt$ and $Nb$. Further, the $R{e}_{x}^{-0.5}S{h}_{x}$ is an increasing property of $Nb$ and a decreasing property of $Nt$. The ${Re}_{\mathrm{x}}^{-0.5}N{u}_{x}$ is a decreasing property of $\phi$ whereas the ${Re}_{\mathrm{x}}^{-0.5}N{u}_{x}$ is an increasing property of ${\beta }_{E}$ (see Fig. 21). Figure 22 designates that the $R{e}_{x}^{-0.5}S{h}_{x}$ is an increasing property of both $\phi$ and $Le$.

## Final remarks

Three-dimensional dynamics of Cu–${\mathrm{H}}_{2}\mathrm{O}$ nanofluid due to a bi-directional stretchable flat film with haphazard motion and thermo-migration of nanoparticles is investigated. The modified Boungirno nanofluid model is implemented by considering the thermo-physical properties of nanofluid. The effects of exponential heat generation and convective heating type boundary conditions are also accounted for. The key results of this analysis are:

- The convective conditions lead to an enhancement of temperature and nanoparticle concentration profiles.
- The velocity ratio factor exhibits decreasing behavior for $x$-component velocity.
- The velocity ratio factor exhibits increasing behavior for $y$-component velocity.
- The higher temperature is noticed for larger values of $Nb$ and $Nt$.
- The temperature field enhances with $\phi$.
- The Lewis number and Brownian motion factor decline the nanoparticle concentration field.
- The rate of heat transport at the wall is reduced for larger values of $Nb$ and $Nt$.
- The mass transport rate improves with $\phi$ and $Le$.

## Figures and Tables

**Figure 23:**
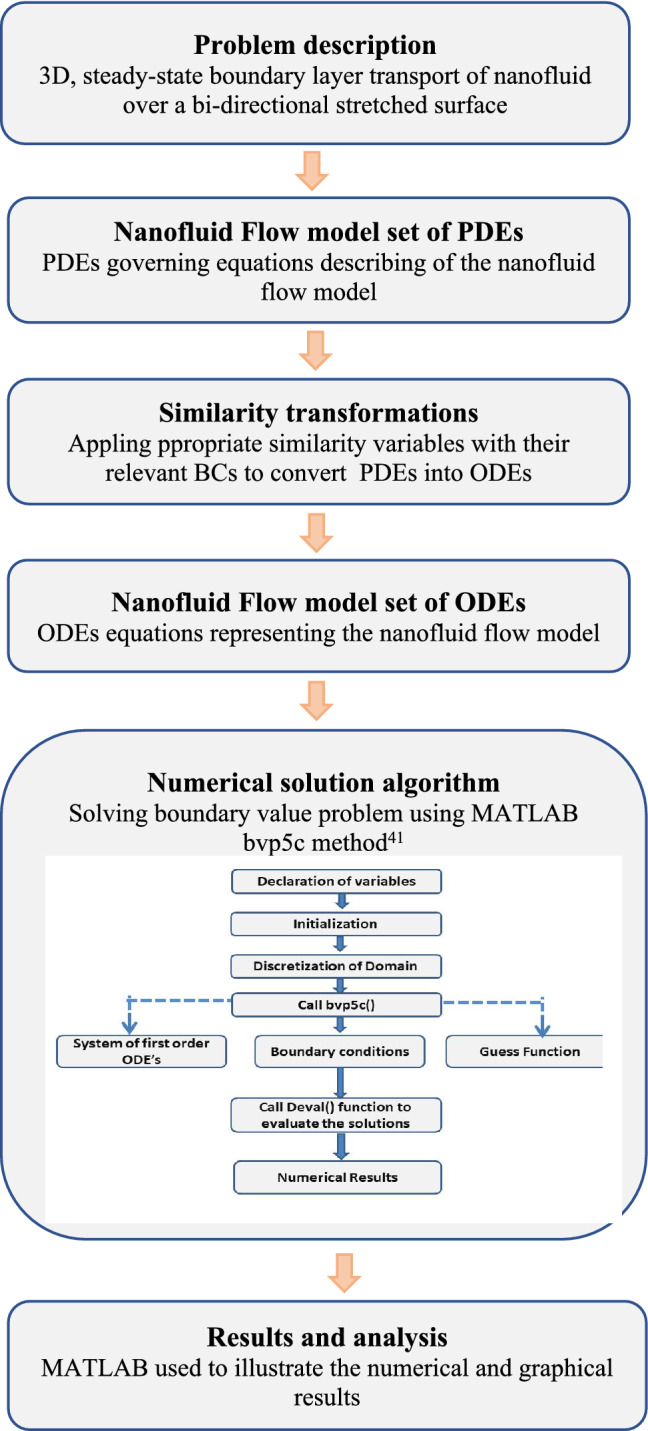
Boundary value problem solution method flow chart with bvp5c^41^ algorithm. At first, the problem is described, then modeled by a set of nonlinear partial differential equations (PEDs) and the boundary layer approximation. The studied problem governing set of PDEs' were non dimensionalized using similarity transformation non-dimensional variables and applied relevant boundary conditions. The obtained set of coupled linear ordinary differential equations (ODEs) representing the problem is solved numerically using the built-in bvp5c technique in computational software MATLAB. Detailed info on the algorithm used at the bvp5c function is described in the appendix on Owhaib et al.^41^ The numerical and graphical results are illustrated using MATLAB. A discussion on the impact of prevailing parameters' is provided.

## Appendix

The solution method flowchart applied to solve the current study boundary value problem is visualized in Fig. 23.﻿﻿ At first, the problem is described, then modeled by a set of nonlinear partial differential equations (PEDs) and the boundary layer approximation. The studied problem governing set of PDEs' were non dimensionalized using similarity transformation non-dimensional variables and applied relevant boundary conditions. The obtained set of coupled linear ordinary differential equations (ODEs) representing the problem is solved numerically using the built-in bvp5c technique in computational software MATLAB. Detailed info on the algorithm used at the bvp5c function is described in the appendix on Owhaib et al.^41^ The numerical and graphical results are illustrated using MATLAB. A discussion on the impact of prevailing parameters' is provided.

## List of symbols

$$a, b$$: Constants
$$Bi$$: Biot number
$C$: Nanoparticle’s volume fraction
${C}_{p}$: Specific heat (J/kg K)
${D}_{B}$: Brownian diffusion coefficient
${D}_{T}$: Thermo-migration diffusion coefficient
$$f$$*, *$$g$$: Transform velocities
$$k$$: Thermal conductivity (W/m K)
$$Le$$: Lewis number
$Nb$: Brownian motion parameter
$$Nt$$: Thermophoresis parameter
$$Nu$$: Nusselt number
$$Pr$$: Prandtl number
$$Re$$: Reynolds number
$$Sh$$: Sherwood number
$$Sf$$: Friction factor
$$T$$: Temperature (K)
${T}_{f}$: Stretched nanofluid film surface temperature (K)
$${T}_{\infty }$$: Ambient temperature (K)
$u, v, w$: Velocities components along $x, y$ and $z$‐directions (m/s)
${U}_{w}\left(x\right), {V}_{w}(y)$: Nanofluid film surface velocities (m/s)

## Greek symbols

$$\alpha$$: Thermal diffusivity (m^2^/s)
${\beta }_{E}$: Exponential heat source space parameter
${\beta }_{e}$: Exponential heat source space coefficient
$$\eta$$: Similarity variable
$\theta$: Transform temperature distribution
$\Theta$: Transform nanoparticles volume fraction distribution
$\lambda$: Stretching ratio parameter
μ: Dynamic viscosity (kg/m s)
ν: Kinematic viscosity (m^2^/s)
ρ: Density (kg/m^3^)
$\phi$: Nanoparticles fraction in the nanofluid by volume

## Subscripts

$$\infty$$: Ambient state
*f*,w: Surface state
$$l$$: Base fluid
$$nl$$: Nanofluid
$$np$$: Nanoparticles
